# Novel C6-substituted 1,3,4-oxadiazinones as potential anti-cancer agents

**DOI:** 10.18632/oncotarget.5839

**Published:** 2015-10-23

**Authors:** Md. Maqusood Alam, Su-Chan Lee, Yujin Jung, Hye Jeong Yun, Hye-Young Min, Ho Jin Lee, Phuong Chi Pham, Jayoung Moon, Dah In Kwon, Bumhee Lim, Young-Ger Suh, Jeeyeon Lee, Ho-Young Lee

**Affiliations:** ^1^ College of Pharmacy and Research Institute of Pharmaceutical Sciences, Seoul National University, Seoul 08826, Korea

**Keywords:** oxadiazinone, insulin-like growth factor 1 receptor, Src, molecular docking analysis, drug resistance

## Abstract

The insulin-like growth factor 1 receptor (IGF-1R) is a membrane receptor tyrosine kinase over-expressed in a number of tumors. However, combating resistance is one of the main challenges in the currently available IGF-1R inhibitor-based cancer therapies. Increased Src activation has been reported to confer resistance to anti-IGF-1R therapeutics in various tumor cells. An urgent unmet need for IGF-1R inhibitors is to suppress Src rephosphorylation induced by current anti-IGF-1R regimens. In efforts to develop effective anticancer agents targeting the IGF-1R signaling pathway, we explored *2-aryl-1,3,4-oxadiazin-5-ones* as a novel scaffold that is structurally unrelated to current tyrosine kinase inhibitors (TKIs). The compound, LL-2003, exhibited promising antitumor effects *in vitro* and *in vivo*; it effectively suppressed IGF-1R and Src and induced apoptosis in various non-small cell lung cancer cells. Further optimizations for enhanced potency in cellular assays need to be followed, but our strategy to identify novel IGF-1R/Src inhibitors may open a new avenue to develop more efficient anticancer agents.

## INTRODUCTION

The insulin-like growth factor 1 receptor (IGF-1R) is a membrane receptor tyrosine kinase implicated in oncogenic transformation and metastasis and is over-expressed in a number of tumors [1–5]. The IGF-1R signaling pathway plays a critical role in the proliferation and survival of various tumor types. The IGF-1R signaling pathway is associated with resistance to many chemotherapeutics, including chemotherapy, radiation, and molecularly targeted therapies, which is of particular importance [1, 6–9]. Therefore, effective regimens to inactivate the IGF-1R pathway have been anticipated to provide clinical benefits to cancer patients [6, 10–12]. Accordingly, the IGF-1R signaling pathway has been a major target for the development of anticancer agents. However, the therapeutic efficacy of IGF-1R inhibitors, mainly monoclonal antibodies (mAb) and tyrosine kinase inhibitors (TKI), has been modest in a variety of human cancers [13–16]. In particular, increased Src activation is thought to be related to the resistance to IGF-1R inhibitor-based anticancer therapies [17, 18]. Combating resistance is one of the main challenges in the currently available IGF-1R inhibitor-based cancer therapies [7]. Combined treatment with IGF-1R and Src inhibitors could result in toxicity problems. Therefore, an urgent unmet need for developing dual inhibitors that target both IGF-1R and Src remains.

Peptoids (oligomers of N-substituted glycines) are a class of peptidomimetics known to interact with various proteins [19–23]. Although they show promise for therapeutic use [24–27], their flexibility and limited hydrogen bonds compared to peptides have limited their therapeutic applications [28–31]. *2-Aryl-4H-1,3,4-oxadiazin-5(6H)-ones*, rigidified forms of peptoids [23, 32–34], can be synthesized using solid phase peptoid synthesis [35, 36]. Few biological effects of these compounds are known other than monoamine oxidase (MAO) inhibition and preliminary anticancer activity [37, 38]. These molecules are chemically unrelated to other current anticancer agents, showing potential as a new chemotherapeutic scaffold. The chemical space of current TKIs has been limited to only small number of scaffolds, including substituted benzene, pyrimidine/pyridine, piperazine/morpholine, or benzamide/phenyl urea. Therefore, a large part of the chemical space is yet to be explored [39–42]. To synthesize novel compounds that can suppress both IGF-1R and Src, we explored new chemical scaffolds, not related to current TKIs, by the construction of libraries of peptoids along with molecular docking studies.

Here, we demonstrate the preclinical evidence of promising antitumor effects of LL-2003, a novel oxadiazinone compound, *in vitro* and *in vivo*. LL-2003 effectively suppressed IGF-1R and Src and induced apoptosis in various non-small cell lung cancer cells. Although further optimizations for enhanced potency in cellular assays need to be followed, the unique activities of the novel oxadiazinone compound that we identified from the peptidomimetic library may lead to new mechanistic studies exploring signaling pathways associated with cancer resistance and may overcome the limitations of current mAbs or TKI inhibitors.

## RESULTS

### Activation of Src in NSCLC cells after treatment with an IGF-1R TKI

**Figure 1:**
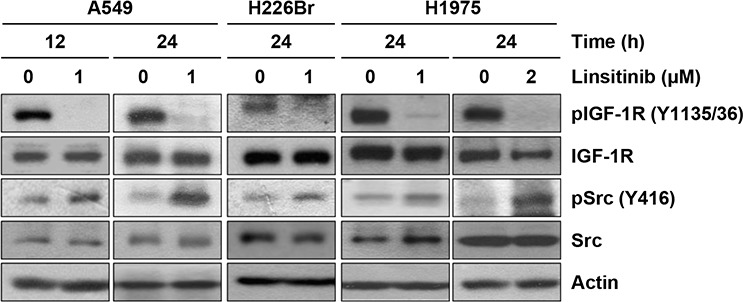
Induction of Src activation by linsitinib treatment A549, H226Br, and H1975 cells were treated with 1 or 2 μM linsitinib (diluted in RPMI 1640 medium containing 10% FBS) for 12 or 24 h. Before harvest, cells were further stimulated with 10% FBS for 20 min. Cells were lysed with modified RIPA buffer and the protein expression was determined by Western blot analysis.

In our previous studies, inhibition of IGF-1R signaling with a monoclonal antibody (cixutumumab) induced Src reactivation as a bypassing mechanism via binding of free IGF1 to integrin β3, resulting in resistance to the drug [17]. In this study, we also observed that treatment with linsitinib (OSI-906), an IGF-1R TKI, also induced a time- and dose-independent Src activation in several non-small cell lung cancer (NSCLC) cell lines, including A549, H226Br, and H1975. As shown in Figure 1, IGF-1R phosphorylation was blocked by treatment with linsitinib for up to 24 h. However, a concomitant Src re-phosphorylation was observed, indicating a reprogramming of the NSCLC cell lines in response to the blockade of IGF-1R signaling by treatment with an IGF-1R TKI.

### Synthesis of C6-functionalized oxadiazinone derivatives

**Figure 2:**
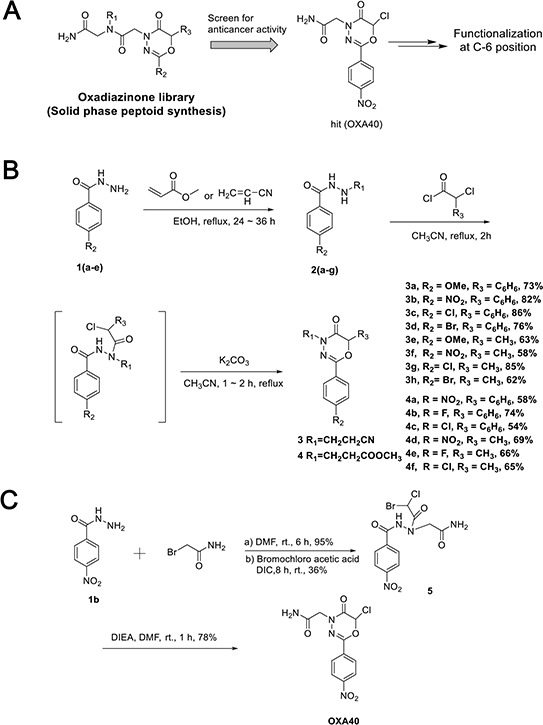
Synthesis of oxadiazinone derivatives **A.** Hit compound (OXA40) was screened for anti-cancer activity from the peptoid library. **B.** Synthesis of *2-aryl-1,3,4-oxadiazin-5-ones* containing functional groups at C6 position. **C.** Synthesis of OXA40 in solution phase.

In order to develop novel anticancer regimens to inhibit the IGF-1R signaling without activating the Src signaling pathway, we have begun an investigation into the discovery of compounds that block IGF-1R activation without a concomitant Src activation. Previous anticancer activity screenings have led us to identify OXA40 as a hit compound for IGF-1R inhibition from the oxadiazinone peptoid (OXA) libraries (Figure 2A). This compound with the –Cl group at C6 was obtained from solid phase peptoid synthesis using MBHA resin. After initial Fmoc deprotection, acylation with bromoacetic acid and N,N’-diisopropylcarbodiimide in DMF was followed by nucleophilic displacement with benzohydrizide for 1 hour. For the last acylation step, bromochloroacetic acid was used to incorporate the –Cl group. The final cyclization reaction was performed using excess N,N-diisopropylethylamine to ensure complete cyclization to afford oxadiazinone compounds.

Other studies of oxadiazinone synthesis have used derivatization only at the N4 or C2 positions for MAO inhibitor development [37]. However, the C6 position of the oxadiazinone scaffold has rarely been functionalized. Our docking analysis with OXA40 suggested that C6 could be functionalized to fit better in the binding site of IGF-1R (Figure S1). When the chlorine atom was replaced with a bulky aromatic group, the docked molecules aligned well with increased docking scores (Table S1). To expand the functionality of the oxadiazinone scaffold, methyl, phenyl, and halogen groups were incorporated at C6 of oxadiazinones as depicted in Figure 2B. Michael addition followed by acylation with α-chlorophenylacetyl chloride or 2-chloropropionyl chloride afforded corresponding benzohydrazide intermediates, which were then subsequently cyclized to generate 3a–3h and 4a–4f. Compound OXA40 was obtained in low yield from the solid phase synthesis, presumably due to acid-promoted ring opening. We also established a new synthesis of OXA40 in solution, where the vigorous cleavage step associated with TFA can be avoided (Figure 2C).

### LL-2003 (3c) displays antiproliferative activities and suppresses IGF-1R and Src phosphorylation in human non-small cell lung cancer cells

**Figure 3:**
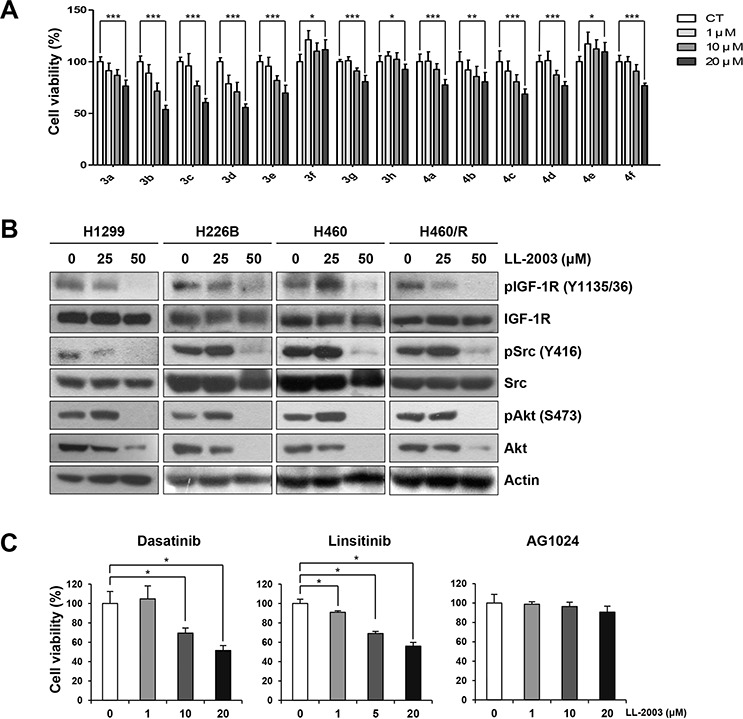
Suppression of cell viability and IGF-1R and Src signaling by treatment with LL-2003 **A.** Cells seeded in 96-well plates were treated with various concentrations of oxadiazinone derivatives for 3 d. Cell viability was determined by the MTT assay. **B.** Western blot analysis of the indicated protein expressions in H1299, A549, H226B, H460, and H460/R cells treated with indicated concentrations of LL-2003. **C.** Cells seeded in 96-well plates were treated with various concentrations of dasatinib, linsitinib, or AG1024 for 3 d. Cell viability was determined by the MTT assay. *P < 0.05; **P < 0.01; ***P < 0.001.

The effect of oxadiazinones 3a–3h and 4a–4f on the viability of non-small cell lung cancer (NSCLC) cells was tested in H1299 cells by the MTT assay (Figure 3A). Among the 14 oxadiazinone derivatives shown in Figure 2B, several compounds displayed concentration-dependent inhibitory effects on cell viability. In particular, LL-2003 (3c) and 3b at the concentration of 20 μM reduced cell viability by ~40 and 46%, respectively. The phenyl substitutions at the C6 position of oxadiazinone resulted in enhanced potency compared to methyl substituents. These C6-substituted derivatives of oxadiazinones moderately inhibited the viability of H1299 cells in a concentration-dependent manner, with the IC_50_ values ranging from 22.4 to 64.6 μM (Table 1). Based on this result, we chose LL-2003 (3c) as a potential IGF-1R inhibitor and evaluated its anticancer activities in various NSCLC cell lines.

**Table 1:** IC_50_ values of selected oxadiazinone derivatives determined by the MTT assay

Compds	R_1_	R_2_	R_3_	IC_50_ (μM)
**OXA40**	−CH_2_CONH_2_	−NO_2_	−Cl	64.6
**3b**	−CH_2_CH_2_CN	−NO_2_	−Ph	35.2
**3c (LL-2003)**	−CH_2_CH_2_CN	−Cl	−Ph	22.4
**3d**	−CH_2_CH_2_CN	−Br	−Ph	51.4
**4c**	−CH_2_CH_2_COOCH_3_	−Cl	−Ph	47.6

We then assessed the effects of LL-2003 on the phosphorylation of IGF-1R and Src. LL-2003 effectively suppressed the phosphorylation of IGF-1R and Src (Figures 3B and S2). Total IGF-1R and Src expressions remained unaffected by the treatment. In addition, the phosphorylation of Akt, a converging downstream of IGF-1R and Src, was also suppressed by LL-2003 treatment. In addition, LL-2003 did not modulate the mRNA expression of IGF-1R and Src at the same experimental conditions (Figure S3). These results suggest that LL-2003 effectively suppresses the phosphorylation of both IGF-1R and Src without affecting total expression levels in NSCLC cells lines. A recent finding showed inactivation of Akt2 not by a Src inhibitor dasatinib but by an IGF-1R inhibitor BMS-754807 [43], suggesting that Akt2 could be activated by the IGF-1R pathway in a Src-independent manner. Based on this notion, we assessed the effect of LL-2003 on the phosphorylation of Akt2. Immunoprecipitation analysis revealed Akt2 phosphorylation was effectively regulated by treatment with LL-2003 (Figure S4). This result suggests that LL-2003 could suppress the IGF-1R signaling pathway through Src-dependent and Src-independent mechanisms. The inhibitory effects of LL-2003 (3c) and 3b on cell viability were comparable to those of clinically available Src (dasatinib) or IGF-1R (linsitinib) TKIs or greater than those of a preclinical IGF-1R inhibitor (AG1024) (Figure 3C).

### The antiproliferative activities of LL-2003 (3c) are mediated by apoptosis in NSCLC cells

**Figure 4:**
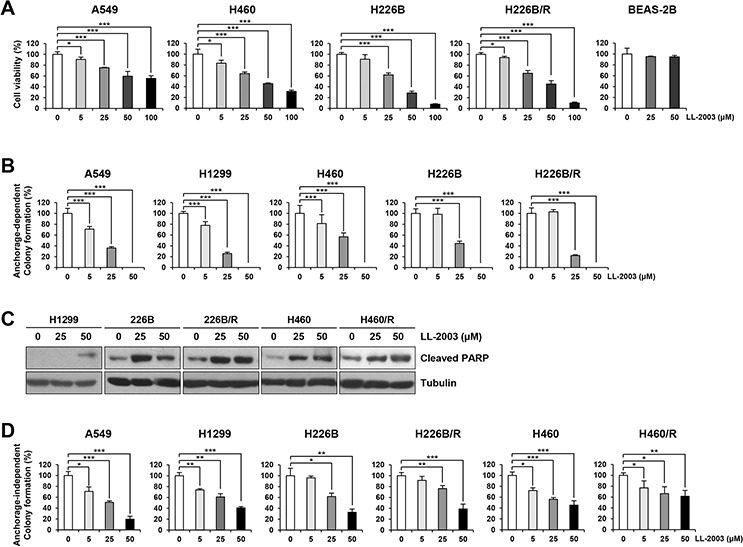
Suppression of cell viability and colony formation and induction of apoptosis by treatment with LL-2003 **A.** The decreases in cell viability by treatment with LL-2003 were examined by the MTT assay. **B.** The inhibitory effect of LL-2003 on the anchorage-dependent colony formation. **C.** NSCLC cells were treated with LL-2003 (25 or 50 μM) for 3 d. Adherent and floating cells were collected and then lysed with modified RIPA buffer. Increases in PARP cleavage, an indicator of apoptosis, were determined by Western blot analysis. **D.** Soft agar assay for determining the effect of LL-2003 on the anchorage-independent colony formation. *P < 0.05; **P < 0.01; ***P < 0.001.

Next, we examined the effects of LL-2003 on the viability and colony forming abilities of various NSCLC cell lines (i.e., A549, H1299, H226B, H226B/R, and H460). The MTT assay revealed that LL-2003 significantly inhibited the viability of NSCLC cells in a concentration-dependent manner (Figure 4A). LL-2003 displayed no obvious effects on the viability of BEAS-2B normal human bronchial epithelial cells (Figure 4A), suggesting minimal toxicity of LL-2003 to normal cells. Anchorage-dependent colony forming abilities of these cell lines were similarly suppressed by the drug treatment (Figure 4B). To investigate the mechanisms by which LL-2003 suppressed the viability and colony formation abilities of NSCLC cells, we examined whether LL-2003 has the capacity to induce apoptosis. Poly-(ADP-ribose) polymerase (PARP) cleavage was markedly increased in H1299, H226B, H226B/R, H460, and H460/R by treatment with LL-2003 in a dose-dependent manner (Figures 4C and S2). These findings suggest that the capacity of a dual IGF-1R/Src inhibitor, LL-2003, to induce apoptosis. We assessed the effects of LL-2003 on anchorage-independent colony formation of NSCLCs using soft agar assay because tumorigenic cells can grow in semisolid medium, especially in soft agar [44]. Treatment with LL-2003 displayed significantly inhibitory effects on colony formation of various NSCLC cells (Figure 4D). LL-2003 exhibited similar effects on cell viability, the phosphorylation of IGF-1R and Src, and induction of PARP cleavage in MCF7 human breast cancer cells, suggesting LL-2003 could be applicable to other types of cancers (Figure S5). These findings indicate *in vitro* evidence supporting the efficacy of dual IGF-1R/Src inhibitor, LL-2003, in NSCLC cells.

### The effects of LL-2003 on tumorigenic activities of NSCLC cells *in vitro* and *in vivo*

**Figure 5:**
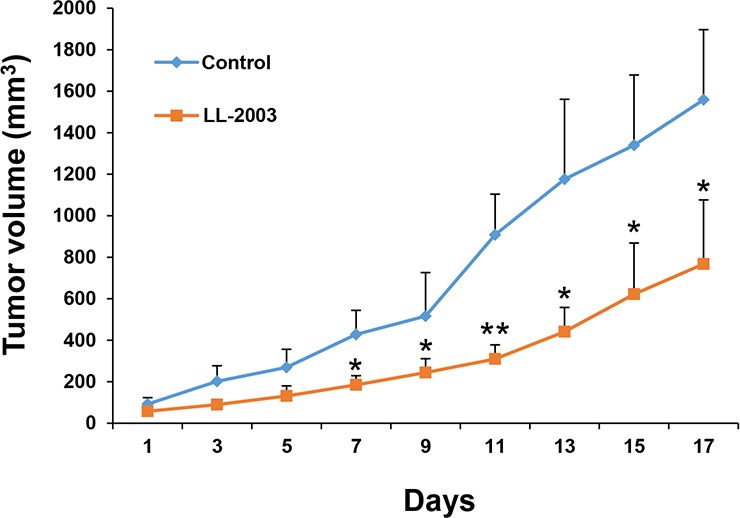
Antitumor effect of LL-2003 in a tumor xenograft model NOD/SCID mice bearing H1299 tumor xenografts were randomly grouped and treated with vehicle or LL-2003 every day for 17 d. The changes of tumor growth were monitored every other day. *P < 0.05; **P < 0.01.

We tested the antitumor activities of LL-2003 in a H1299 xenograft tumor model. Mice bearing tumors that reached a volume of 50–150 mm^3^ were daily treated with vehicle (20 μL of DMSO and 480 μL of corn oil), or LL-2003 (50 mg/kg body weight) by intraperitoneal injection. Treatment with LL-2003 for 17 d significantly inhibited tumor growth compared to the control group, indicating effective antitumor activities of LL-2003 *in vivo* (Figures 5 and S6). During the treatment, body weight was not significantly changed. Moreover, tissue samples obtained from several organs (liver, lung, heart, kidneys, spleen, urinary bladder, ovary, stomach, pancreas, colon, and rectum) of the LL-2003-treated mice revealed no remarkable histopathological changes, suggesting that LL-2003 is minimally toxic in mice. These results indicate the potential of LL-2003 as an anticancer drug against NSCLC cells.

### Molecular docking studies to predict possible mode of binding

To examine possible interactions of the compounds in the binding site, we used the published crystal structure of IGF-1R complexed with the inhibitor PQIP (PDB:3D94) for the docking study [45]. Due to the well-overlaid curvatures of these compounds to the reported ligands, we focused on docking the molecules to the ATP binding site. Re-docking of PQIP to the ATP binding site gave a pose similar to the original X-ray structure (0.579 Å), which validates the docking method. As a result, docking scores increased when a phenyl group was added to the oxadiazinone ring (Table S1).

**Figure 6:**
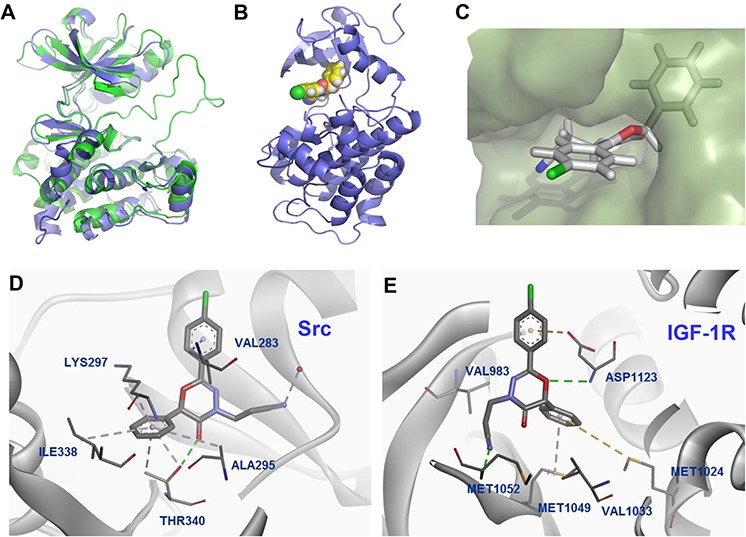
Predicted binding mode from docking analysis **A.** Overlaid structures of IGF-1R (PDB: 3D94; green) [45] and Src (PDB: 1YOL; purple) [46]. **B.** The orthogonal view of 3c bound in the binding site of Src. **C.** Surface diagram of Src with the bound structure of 3c. **D.** Interaction diagram of 3c with Src residues (PDB: 1YOL). **E.** Docked poses of 3c, exhibiting additional hydrophobic interactions in the binding site of IGF-1R (PDB: 3D94). Each color represents hydrogen bond (green), -sulfur (gold), -alkyl hydrophobic interaction (pink).

As shown in Figure 6A, human Src (pdb: 1YOL) and IGF-1R (PDB: 3D94) have high homology in their overlaid structures. These two proteins share an overall fold of 8 α-helices and 1 β-sheet with an overall RMSD of 6.375 Å (aligned based on the Cα atoms of each residue), despite only 39% homology in amino acid sequence. LL-2003 aligned well within the ATP binding pocket of Src [46]. The -Cl group is oriented toward the surface, while the phenyl group fits deeply into the hydrophobic pocket (Figure 6B and 6C) [47]. The C6 phenyl substituent on the oxadiazinone ring fits into a hydrophobic pocket comprised of Ala295, Ile338, and Thr340, resulting in improved docking scores compared to OXA40 (Figure 6D and Table S1). Thr340, a hinge residue for Src specificity, has a hydrogen bond with the carbonyl oxygen of LL-2003 in addition to a π-alkyl interaction with the phenyl group of LL-2003. The side chain of Val283, part of the Gly loop in Src, has formed a hydrophobic interaction with the phenyl ring of LL-2003.

The docking result with IGF-1R shown in Figure 6E also suggests that the phenyl group of oxadiazinone has hydrophobic interactions with the N lobe residues (Met1024 and Met1049), contributing to the stabilization of LL-2003 in the binding site. In particular, Met1024 formed a favorable π-sulfur interaction with the phenyl group at the C6 position [48]. Met1049, the gatekeeper residue of IGF-1R, also has a π-alkyl interaction with the phenyl group at C6. The O1 atom of LL-2003 has a hydrogen bond with Asp1123 which is a part of the conserved ‘DFG motif’ in kinases. As shown above, our docking analyses suggest the possible binding mode of LL-2003, demonstrating that the aromatic group at C6 of the oxadiazinone ring is important for both IGF-1R and Src inhibitions.

### Interactions of LL-2003 with the hinge binding region of IGF-1R/Src proteins

Molecular model analyses along with the sequence alignment of Src and IGF-1R showed that the hinge region of both kinases have critical interactions with LL-2003 (Figure 6D and 6E). The amide proton of Met1052 (conserved hinge residue of IGF-1R) has a hydrogen bond with the nitrile group of LL-2003 in IGF-1R (pdb: 3D94). Threonine 340, part of the hinge region of Src, was reported to interact with the known ligand (CGP77675) via a hydrogen bond [46]. This residue, known to be important for Src selectivity, retains a hydrogen bond with the carbonyl oxygen of the oxadiazinone ring (Figure 6D, shown as a green line). The data are consistent with the common hinge binding contribution of kinase inhibitors, suggesting dual inhibition through favorable hinge binding for both kinases.

## DISCUSSION

Numerous studies have shown the plasticity of tumor cells in which the obstruction of particular targets leads to activation of multiple compensatory signaling pathways, resulting in an adaptive survival of tumor cells and thus induction of drug resistance. We have shown involvement of Src, which acts as a common downstream node of multiple signaling pathways initiated by membrane-associated receptors, in resistance to IGF-1R inhibitors. The studies reported herein demonstrate that LL-2003, a derivative of *2-aryl-1,3,4-oxadiazin-5-ones*, has promising anticancer activities at least in part by blocking both IGF-1R and Src (Figure S7). Our results provide pre-clinical support for the use of LL-2003 as a dual IGF-1R and Src in the treatment of lung cancer.

IGF-1R-targeted therapies have been anticipated as a promising single-agent or combination regimen. The canonical activation of IGF-1R stimulated by the binding of ligands to IGF-IR [12], leads to the autophosphorylation of tyrosine residues 1131, 1135, and 1136 in the activation loop of the IGF-1R. Src, a non-receptor protein tyrosine kinase, has been found to directly induce IGF-1R phosphorylation [49]. Since Src and IGF-1R are coactivated in most NSCLC cell lines and tumors in patients [18] and plays an important role in cancer cell survival and resistance to targeted anticancer therapies [50, 51], and cancer cells appear to display resistance to the IGF-1R-targeted agents through Src activation [17, 18], we postulated that dual targeting of IGF-1R and Src could be an effective way to overcome anticancer drug resistance and dual IGF-1R/Src TKI regimens may offer a novel opportunity for cancer therapy. In support of the notion, our previous studies clearly revealed the potent antitumor activities of co-targeting IGF-1R and Src in various NSCLC cells *in vitro* and *in vivo* [17, 18].

Although these results show the effectiveness of combined treatment of IGF-1R and Src inhibitors in NSCLC treatment, the main drawbacks of these strategies are undesirable side effects. It is necessary to develop a novel dual inhibitor that targets IGF-1R and Src. Hence, our approach to develop dual IGF-1R/Src TKI began with exploring 2-aryl-4H-1,3,4-oxadiazin-5(6H)-one as a novel scaffold that is structurally unrelated to current TKIs. The majority of small-molecular weight inhibitors of tyrosine kinases have displayed limited variety in chemical structures [52]. Famous examples are a urea for sorafenib, 4-anilino-quinazolines for gefitinib and erlotinib, and a phenyl-amino-pyrimidine for imatinib. In addition, an efficient strategy to combat the emerging drug-resistance in cancer therapy has been found to develop a new scaffold whose chemical structure and mode of binding are distinct from previous drugs. Imatinib and nilotinib contain a phenyl-amino-pyrimidine, while dasatinib, used for imatinib-resistant CML, possesses an amino-thiazole moiety. Sunitinib, used for imatinib-resistant gastrointestinal stromal tumors (GIST), has an indolinone unit.

Kinases are one of the most intensively pursued drug targets, with almost 30,000 kinase inhibitors having been reported in the public database [41]. Recently, enormous efforts have been made to identify selectivity determinants in kinases: inactive conformations, allosteric sites, cysteine residues for irreversible binding, and the gate keeper residues [53]. Out of 196,904 potential hinge binding fragments retrieved from ZINC database, only 1% of those motifs are covered by kinase inhibitors [41]. Frequently used hinge binding motifs of kinases are pyrimidine, pyridine, piperazine, and morpholine. Phenol is preferred to generate type I_1/2_ inhibitors. These findings suggest that a large part of chemical space has not yet been explored. Toward this aim, we investigated oxadiazinone derivatives to discover new heterocyclic compounds for IGFR inhibition.

We developed a novel oxadiazinone scaffold from our druggable peptoid library and improved anticancer activity based on molecular docking analysis. Among them, LL-2003 demonstrated promising antitumor activities along with dual inhibition of IGF-1R and Src. We found several features of LL-2003 that place it as a lead compound for the development of novel anticancer drugs. First, LL-2003 significantly inhibited the proliferative activity in a panel of NSCLC cell lines and their sublines with acquired chemoresistance cells. LL-2003 also exhibited antitumor activity in a xenograft tumor model bearing H1299 NSCLC cells. These findings indicate the potential of LL-2003 for both first-line and second-line therapies. Second, LL-2003 appeared to induce apoptosis in several NSCLC cell lines. LL-2003 was derived from the peptoid-based library, which may result in a distinct signaling indication compared to current TKIs. Current IGF-IR mAb and TKIs do not induce apoptosis while reducing cell viability [54]. We have demonstrated induction of apoptosis in NSCLC cells by the combined treatment with IGF-1R and Src inhibitors [17, 18]. Consequently, a compound that targets both IGF-1R and Src is expected to induce apoptosis. In line with the same notion, dual inhibition of IGF-1R and Src by the treatment with LL-2003 appeared to induce apoptosis in NSCLC cells. Third, the rapid synthesis of LL-2003 within 3 steps can furnish a good synthetic platform for a large library for enhanced potency. Convenient synthesis of a large library along with facile derivatization means that our oxadiazinone compounds represent a new chemical scaffold for the development of anticancer agents. Enormous efforts in drug discovery have been directed toward controlling apoptotic signals since the effectiveness of anticancer drugs is associated with apoptosis in many cases. Many protein-protein interaction modulators mimic intrinsic apoptosis-modulating peptides with enhanced stability to peptidases and are expected to be effective in apoptosis signaling pathways [55]. There are many examples of peptide-based therapeutics that target apoptosis [56, 57]. In particular, constrained peptoids were found to control apoptosis [31, 58]. As such, the peptoid-based library could provide the platform for the development of novel anticancer drugs. These data show that LL-2003 could be a potential anticancer drug targeting both IGF-1R and Src.

Taken together, our findings provide the potential of LL-2003 as an effective anticancer agent targeting both IGF-1R and Src in human NSCLC. Many peptidomimetics are developed for their anticancer effects [59–62], but our work is the first example of peptoid-based anticancer agents to our knowledge. The knowledge obtained in this work may be incorporated to design better anticancer agents, providing valuable tools to understand drug resistance mechanisms in cancer therapy.

## MATERIALS AND METHODS

### General instrumentation and chemicals

All solvents were purified and used in scrupulously dry conditions. NMR spectra of all new compounds were recorded on JEOL JLM-LA 300, Varian Gemini 2000, Bruker AVANCE 500 operating at 300 MHz for ^1^H and 125 MHz for ^13^C NMR. Chemical shifts (δ) are reported in ppm, downfield from the internal TMS standard. Analytical thin layer chromatography (TLC) was carried out using precoated silica gel (E. Merck Kiesegel 60F254, layer thickness 0.25 mm), and chromatography was performed using silica gel 60 (40–60 μm).

### General procedure for the synthesis of 3a-3h, and 4a-4f

To a solution of aryolhydrazine 2a-2g (1 mmol) in acetonitrile, α-chlorophenylacetyl chloride (or 2-chloropropionyl chloride) (1.2 mmol) was added dropwise at room temperature. The mixture was refluxed for 2 h. After cooling, anhydrous potassium carbonate was added and suspension was refluxed for 1–2 h. The hot mixture was filtered and the solvent was evaporated in *vacuo* to give oil, which, on cooling, solidified slowly. The residue was purified by column chromatography (EtOAc/*n*-hexane) to give compounds 3a-3h, and 4a-4f. Detailed experimental procedures and characterization of all new compounds and modeling data are available in supplementary information.

### Reagents

Dimethyl sulfoxide (DMSO), crystal violet, 3-(4,5-dimethylthiazol-2-yl)-2,5-diphenyltetrazolium bromide (MTT), pacliaxel, corn oil, and chemicals unless otherwise indicated were purchased from Sigma-Aldrich (St. Louis, MO, USA). Antibodies against pIGF-1R (Y1135/36), IGF-1R, pSrc (Y416), Src, pAkt (S473), Akt, and β-tubulin were purchased from Cell Signaling Technology (Danvers, MA, USA). Antibody against cleaved PARP was purchased from BD Biosciences (San Jose, CA, USA). Antibodies against Akt2 and actin were purchased from Santa Cruz Biotechnology (Santa Cruz, CA, USA). Linsitinib was purchased from Selleckchem (Houston, TX, USA).

### Cell culture

H1299, H460, H1975, and A549 cells were purchased from American Type Tissue Collection (ATCC, Manassas, VA, USA). H226B and H226Br cells were kindly provided by Dr. John V. Heymach (The University of Texas M. D. Anderson Cancer Center, Houston, TX, USA). Cells were cultured in RPMI 1640 (Welgene Inc., Gyeongsan-si, Republic of Korea) supplemented with 10% fetal bovine serum (FBS) and antibiotics. Cells were maintained at 37°C in a humidified atmosphere with 5% CO_2_. To generate paclitaxel-resistant cells (H460/R and H226B/R cells), H460 and H226B cells were continuously exposed to increasing concentrations of paclitaxel for more than 6 months.

### Western blot analysis

Cell lysates were obtained by modified RIPA lysis buffer [50 mM Tris-HCl (pH 7.4), 150 mM NaCl, 1 mM EDTA, 0.25% Sodium deoxycholate, 1% Triton X-100, protease inhibitor cocktail (Roche Applied Science, Indianapolis, IN, USA), and phosphatase inhibitor cocktail (Roche)]. The lysates were centrifuged at 13,000 rpm for 20 min at 4°C. Supernatants were collected, and protein concentration was determined by BCA assay (Thermo Fisher Scientific, Waltham, MA, USA). 20 μg of proteins were subjected to 8–10% SDS-PAGE. Separated proteins were transferred onto a PVDF membrane (Bio-Rad Laboratories, Hercules, CA, USA). Membranes were blocked with blocking buffer [3% BSA in Tris-buffered saline (TBS) containing 0.1% Tween-20 (TBST)] for 1 h at room temperature and followed by incubation with primary antibodies diluted in blocking buffer overnight at 4°C. After washing three times with TBST, membranes were incubated with the secondary antibodies for 1 h at room temperature. Membranes were washed three times with TBST and were visualized using an enhanced chemiluminescence (ECL) detection kit (Thermo Fisher Scientific).

### MTT assay

To assess the effect of oxadiazinone derivatives on the viability of NSCLC cells, the MTT assay was performed as described previously [63, 64]. Cells (1–2 × 10^3^ cells/well in 96-well plates) were treated with increasing concentrations of the compounds for 3 d. Cells were treated with MTT solution (final concentration of 200 μg/ml) and incubated for 2–4 h at 37°C. The formazan products were dissolved in DMSO, and the absorbance was measured at 570 nm. The data are presented as a percentage of the control group.

### Anchorage dependent colony formation assay

Cells were seeded into 6-well plates at a density of 300–500 cells/well. After 24 h, cells were treated with LL-2003 (0, 5, 25, and 50 μM). After 14 d, cells were fixed with 100% methanol for 5 min at room temperature. Cells were further stained with crystal violet solution (0.002%) for 2 h, and then washed with water. Stained colonies were photographed and counted.

### Soft agar assay

Harvested cells were diluted at a density of 1.5–3 × 10^3^ cells/well and mixed with 0.4% agar. The mixture was poured on a 1% bottom agar in 24-well plates. After treatment with LL-2003 for 2–3 weeks, the colonies were stained by MTT solution and were counted.

### Animal experiment

All animal procedures were performed using a protocol approved by the Seoul National University Institutional Animal Care and Use Committee. For the xenograft experiment, H1299 cells (4 × 10^6^ cells/mouse) were subcutaneously injected into a 6-weeks-old NOD/SCID mouse. After the tumor volume reached 50–150 mm^3^, mice were randomly grouped and intraperitoneally administered with vehicle (10% DMSO in corn oil) or LL-2003 (50 mg/kg) every day for 17 d. Tumor growth was determined by measuring the short and long diameters of the tumor with a caliper. Body weight was measured to monitor toxicity. The tumor volume was calculated using the following formula: tumor volume (mm^3^) = (short diameter)^2^ × (long diameter) × 0.5.

### Molecular docking analysis

Molecular docking studies were carried out using the Surflex-Dock in Sybyl-X2.1.1 (Tripos Inc, St Louis, MO) with the crystal structures of IGF-1R and Src complexed with ligands (PDB: 3D94 for IGF-1R and 1YOL for Src). The new ligands were prepared by generating 3D conformations from 2D structures using Concord. The protein was energy-minimized using conjugate gradient minimization (Powell's method) until the RMSD was lower than 0.001 kcal/mol·Å. The original ligand was removed and water molecules were also removed unless involved in hydrogen bonding. Docking simulation was carried out using Surflex-Dock GeomX mode. The protomol was usually generated using a threshold of 0.5 Å and bloat of 0 Å. Docking performance was validated by examination of the RMSD of the re-docked structure compared to the original pose. Molecular interactions between ligand and protein were further analyzed using Discovery studio 4.0 Visualizer.

### Statistics

Data are presented as the mean ± SD. All *in vitro* experiments were independently performed at least twice, and a representative result is presented unless otherwise indicated. The statistical significance was analyzed using two-sided Student's *t*-test using Microsoft Excel 2013 (Microsoft Corp., Redmond, WA, USA). *P* values less than 0.05 were considered statistically significant.

## SUPPLEMENTARY DATA FIGURES AND TABLE


