# Supplementary material for: Novel C6-substituted 1,3,4-oxadiazinones as potential anti-cancer agents
Source: Oncotarget. 2015 Oct 23;6(38):40598–610. doi: 10.18632/oncotarget.5839 (PMC4747355; doi:10.18632/oncotarget.5839)
Supplement: Supplementary file 1 [file oncotarget-06-40598-s001.pdf]

SUPPLEMENTARY DATA

EXPERIMENTAL SECTION

Synthesis of 3a-3h and 4a-4f

To a solution of aryolhydrazine **2a-2g** (1 mmol) in acetonitrile, α-chlorophenylacetyl chloride (or 2-chloropropionyl chloride) (1.2 mmol) was added dropwise at room temperature. The mixture was refluxed for 2 h. After cooling, anhydrous potassium carbonate was added and suspension was refluxed for 1-2 h. The hot mixture was filtered and the solvent was evaporated in *vacuo* to give oil, which, on cooling, solidified slowly. The residue was purified by column chromatography (EtOAc/*n*-hexane) to give compounds **3a-3h**, and **4a-4f**.

3-(2-(4-methoxyphenyl)-5-oxo-6-phenyl-5,6-dihydro-4*H*-1,3,4-oxadiazin-4-yl) propanenitrile (3a)

The compound **3a** (120 mg) was obtained according to the general procedure from compound **2a** (100 mg, 0.45 mmol), α-Chlorophenylacetyl chloride (92 µL, 0.54 mmol) and K_2CO_3 (621 mg, 4.5 mmol). Yield 73 %; TLC R_f = 0.40 (EtOAc/*n*-hexane = 1:4); IR (Neat, ν cm^{-1}): 2251.49, 1681.62, 1631.48; ^1H NMR (300 MHz, CD_3OD) δ 7.82 (d, *J* = 7.2 Hz, 2H), 7.52-7.28 (m, 5H), 6.94 (d, *J* = 8.4 Hz, 2H), 5.91 (s, 1H), 4.17 - 4.06 (m, 2H), 3.81 (s, 3H), 2.96-2.91 (m, 2H). ^{13}C NMR (300 MHz, DMSO-d_6): δ 161.6, 159.6, 148.2, 134.9, 129.3, 128.8 (2C), 128.2 (2C), 127.1 (2C), 121.4, 118.8, 114.1 (2C), 75.9, 55.3, 42.5, 15.8.; ESI-MS *m/z* 336 (M + H)$^+$; HR-MS (FAB) *m/z* 336.1339 (calcd for $C_{19}H_{18}N_3O_3$ [M+H]$^+$, 336.1348); HPLC flow rate: 1 mL/min, relative purity 79%, (t = 13.2 min).

3-(2-(4-nitrophenyl)-5-oxo-6-phenyl-5,6-dihydro-4*H*-1,3,4-oxadiazin-4-yl)propanenitrile (3b)

The compound **3b** (98 mg) was obtained according to the general procedure from compound **2b** (80 mg, 0.34 mmol), α-Chlorophenylacetyl chloride (70 µL, 0.40 mmol) and K_2CO_3 (469 mg, 3.4 mmol). Yield 82%; TLC R_f = 0.40 (EtOAc/*n*-hexane = 1:4); IR (Neat, ν cm^{-1}): 2251.49, 1687.41, 1639.30, 1522.52; ^1H NMR (300 MHz, CD_3OD) δ 8.29 (d, *J* = 9.3 Hz, 2H), 8.10 (d, *J* = 9.3 Hz, 2H), 7.50-7.40 (m, 5H), 6.04 (s, 1H), 4.26 - 4.10 (m, 2H), 3.01-2.95 (m, 2H).; ^{13}C NMR (300 MHz, DMSO-d_6): δ 159.5, 148.7, 146.0, 134.9, 134.7, 129.5, 128.9 (2C), 127.5 (2C), 127.3 (2C), 123.9 (2C), 118.7, 76.4, 42.8, 15.9.; ESI-MS *m/z* 351 (M + H)$^+$; HR-MS (FAB) *m/z* 351.1089 (calcd for $C_{18}H_{15}N_4O_4$ [M+H]$^+$, 351.1093); HPLC flow rate: 1 mL/min, relative purity 100 %, (t = 13.4 min).

3-(2-(4-chlorophenyl)-5-oxo-6-phenyl-5,6-dihydro-4*H*-1,3,4-oxadiazin-4-yl)propane- nitrile (3c)

The compound **3c** (116 mg) was obtained according to the general procedure from compound **2c** (90 mg, 0.40 mmol), α-Chlorophenylacetyl chloride (82 µL, 0.48 mmol) and K_2CO_3 (552 mg, 4.0 mmol). Yield 86%; TLC R_f = 0.40 (EtOAc/*n*-hexane = 1:4); IR (Neat, ν cm^{-1}): 2251.49, 1685.48, 1637.27, 750.17; ^1H NMR (300 MHz, CD_3OD) δ 7.89-7.85 (m, 2H), 7.49-7.39 (m, 7H), 5.98 (s, 1H), 4.22-4.07 (m, 2H), 2.99-2.94 (m, 2H).; ^{13}C NMR (400 MHz, DMSO-d_6) δ 159.6, 147.0, 136.0, 134.8, 129.4, 128.87 (2C), 128.84 (2C), 128.1 (2C), 128.0, 127.2 (2C), 118.7, 76.1, 42.6, 15.8.; ESI-MS *m/z* 340 (M + H)$^+$; HR-MS (FAB) *m/z* 340.0861 (calcd for $C_{18}H_{15}ClN_3O_2$ [M+H]$^+$, 340.0853); HPLC flow rate: 1 mL/min, relative purity 95%, (t = 14.6 min).

3-(2-(4-bromophenyl)-5-oxo-6-phenyl-5,6-dihydro-4*H*-1,3,4-oxadiazin-4-yl)propane- nitrile (3d)

The compound **3d** (76 mg) was obtained according to the general procedure from compound **2d** (70 mg, 0.26 mmol), α-Chlorophenylacetyl chloride (53 µL, 0.31 mmol) and K_2CO_3 (358 mg, 2.6 mmol). Yield 76%; TLC R_f = 0.40 (EtOAc/*n*-hexane = 1:4); IR (Neat, ν cm^{-1}): 2251.49, 1685.48, 1637.27, 699.06; ^1H NMR (300 MHz, CD_3OD) δ 7.80-7.76 (m, 2H), 7.61-7.56 (m, 2H), 7.47-7.38 (m, 5H), 5.97 (s, 1H), 4.23-4.05 (m, 2H), 2.95-2.92 (m, 2H).; ^{13}C NMR (300 MHz, DMSO-d_6) δ 159.6, 147.1, 134.8, 131.7 (2C), 129.4, 128.8 (2C), 128.4, 128.2 (2C), 127.2 (2C), 124.9, 118.7, 76.1, 42.6, 15.8.; ESI-MS *m/z* 384 (M + H)$^+$; HR-MS (FAB) *m/z* 384.0353 (calcd for $C_{18}H_{15}BrN_3O_2$ [M+H]$^+$, 384.0348); HPLC flow rate: 1 mL/min, relative purity 100 %, (t = 14.8 min).

3-(2-(4-methoxyphenyl)-6-methyl-5-oxo-5,6-dihydro-4*H*-1,3,4-oxadiazin-4-yl)propanenitrile (3e)

The compound **3e** (62 mg) was obtained according to the general procedure from compound **2a** (80 mg, 0.36 mmol), 2-chloropropinyl chloride (45 µL, 0.43 mmol) and K_2CO_3 (496 mg, 3.6 mmol). Yield 63%; TLC R_f = 0.40 (EtOAc/*n*-hexane = 1:4); IR (Neat, ν cm^{-1}): 2251.49, 1682.59, 1629; ^1H NMR (300 MHz, CD_3OD) δ 7.83 (d, *J* = 6.6 Hz, 2H), 6.97 (d, *J* = 7.2 Hz, 2H), 4.91 (q, *J* = 6.9 Hz, 1H), 4.08-3.97 (m, 2H), 3.83 (s, 3H), 2.92-2.87 (m, 2H), 1.57 (d, *J* = 5.4 Hz, 3H).; ^{13}C NMR (400 MHz, DMSO-d_6) δ 162.1, 161.5, 148.6, 128.2 (2C), 121.6, 118.7, 113.9 (2C), 71.2, 55.3, 42.1, 16.2, 15.8.;

ESI-MS *m/z* 274 (M + H)$^+$; HR-MS (FAB) *m/z* 274.1191 (calcd, for $C_{14}H_{16}N_3O_3$ [M+H]$^+$, 274.1192); HPLC flow rate: 1 mL/min, relative purity 100 %, (t = 11.8 min).

3-(6-methyl-2-(4-nitrophenyl)-5-oxo-5,6-dihydro-4*H*-1,3,4-oxadiazin-4-yl)propanenitrile (3f)

The compound **3f** (71 mg) was obtained according to the general procedure from compound **2b** (100 mg, 0.42 mmol), 2-chloropropinyl chloride (53 µL, 0.50 mmol) and K_2CO_3 (579 mg, 4.2 mmol). Yield 58%; TLC R_f = 0.40 (EtOAc/*n*-hexane = 1:4); IR (Neat, ν cm^{-1}): 2252.45, 1680.66, 1639.20, 1515.78; ^{1}H NMR (300 MHz, CD_3OD) δ 8.30 (d, *J* = 9.2 Hz, 2H), 8.13 (d, *J* = 9.2 Hz, 2H), 5.04 (q, *J* = 6.9 Hz, 1H), 4.13-4.03 (m, 2H), 2.93 (t, *J* = 6.0 Hz, 2H), 1.63 (d, *J* = 6.9 Hz, 3H).; ^{13}C NMR (400 MHz, DMSO-d_6) δ 161.9, 148.7, 146.4, 135.2, 127.5 (2C), 123.8 (2C), 118.6, 71.7, 42.5, 16.4, 15.9.; ESI-MS *m/z* 289 (M + H)$^+$; HR-MS (FAB) *m/z* 289.0945 (calcd, for $C_{13}H_{13}N_4O_4$ [M+H]$^+$, 289.0937); HPLC flow rate: 1 mL/min, relative purity 75%, (t = 11.9 min).

3-(2-(4-chlorophenyl)-6-methyl-5-oxo-5,6-dihydro-4*H*-1,3,4-oxadiazin-4-yl)propane- nitrile (3g)

The compound **3g** (137 mg) was obtained according to the general procedure from compound **2c** (130 mg, 0.58 mmol), 2-chloropropinyl chloride (88 µL, 0.69 mmol) and K_2CO_3 (800 mg, 5.8 mmol). Yield 85%; TLC R_f = 0.40 (EtOAc/*n*-hexane = 1:4); IR (Neat, ν cm^{-1}): 2251.49, 1686.44, 1635.34, 749.20; ^{1}H NMR (300 MHz, CD_3OD) δ 7.85 (d, *J* = 7.5 Hz, 2H), 7.56 (d, *J* = 8.7 Hz, 2H), 4.96 (q, *J* = 7.0 Hz, 1H), 4.14-3.94(m, 2H), 2.93-2.88(m, 2H), 1.59 (d, *J* = 6.8 Hz, 3H).; ^{13}C NMR (400 MHz, DMSO-d_6) δ 162.0, 147.5, 135.9, 128.7 (2C), 128.2, 128.1 (2C), 118.7, 71.5, 42.3, 16.4, 15.9.; ESI-MS *m/z,* 278 (M + H)$^+$; HR-MS (FAB) *m/z* 278.0695 (calcd, for $C_{13}H_{13}ClN_3O_2$ [M+H]$^+$, 278.0696); HPLC flow rate: 1 mL/min, relative purity 95 %, (t = 13.1 min).

3-(2-(4-bromophenyl)-6-methyl-5-oxo-5,6-dihydro-4*H*-1,3,4-oxadiazin-4-yl)propane- nitrile (3h)

The compound **3h** (52 mg) was obtained according to the general procedure from compound **2d** (70 mg, 0.26 mmol), 2-chloropropinyl chloride (40 µL, 0.31 mmol) and K_2CO_3 (358 mg, 2.6 mmol). Yield 62%; TLC R_f = 0.40 (EtOAc/*n*-hexane = 1:4); IR (Neat, ν cm^{-1}): 2251.49, 1686.44, 1633.41, 748.245; ^{1}H NMR (300 MHz, CD_3OD); δ 7.81 (d, *J* = 6.9 Hz, 2H), 7.62 (d, *J* = 6.9 Hz, 2H), 4.95 (q, *J* = 6.9 Hz, 1H), 4.12 - 3.97 (m, 2H), 2.91 (t, *J* = 7.5 Hz, 2H), 1.60 (d, *J* = 6.6 Hz, 3H).; ^{13}C NMR (400 MHz, DMSO-d_6) δ 162.0, 147.5, 131.6 (2C), 128.6, 128.3 (2C), 124.7, 118.6, 71.5, 42.3, 16.3, 15.8.; ESI-MS *m/z,* 322 (M + H)$^+$; HR-MS (FAB) *m/z* 322.0187 (calcd, for $C_{13}H_{13}BrN_3O_2$ [M+H]$^+$, 322.0191); HPLC flow rate: 1 mL/min, relative purity 100 %, (t = 13.4 min).

Methyl 3-(2-(4-nitrophenyl)-5-oxo-6-phenyl-5, 6-dihydro-4*H*-1,3,4-oxadiazin-4-yl) propanoate (4a)

The compound **4a** (82 mg) was obtained according to the general procedure from compound **2e** (100 mg, 0.37 mmol), α-Chlorophenylacetyl chloride (76 µL, 0.45 mmol) and K_2CO_3 (510 mg, 3.7 mmol). Yield 58%; TLC R_f = 0.40 (EtOAc/*n*-hexane = 1:4); IR (Neat, ν cm^{-1}): 1737.55, 1685.48, 1637.27, 1523.49; ^{1}H NMR (300 MHz, CD_3OD); δ 8.26 (d, *J* = 6.9 Hz, 2H), 8.06 (d, *J* = 7.2 Hz, 2H), 7.45 -7.38 (m, 5H), 5.97 (s, 1H), 4.27 - 4.06 (m, 2H), 3.64 (s, 3H), 2.84-2.79 (m, 2H).; ^{13}C NMR (400 MHz, DMSO-d_6) δ 171.2, 159.2, 148.6, 145.6, 135.0, 134.9, 129.4, 128.9 (2C), 127.3 (2C), 127.2 (2C), 123.9 (2C), 76.3, 51.5, 43.2, 31.8.; HR-MS (FAB) *m/z* 406.1015 (calcd for $C_{19}H_{17}N_3O_6$ Na [M+H+Na]$^+$, 406.1015); HPLC flow rate: 1 mL/min, relative purity 95%, (t = 14.1 min).

Methyl 3-(2-(4-fluorophenyl)-5-oxo-6-phenyl-5, 6-dihydro-4*H*-1,3,4-oxadiazin-4-yl)propanoate (4b)

The compound **4b** (167 mg) was obtained according to the general procedure from compound **2f** (150 mg, 0.62 mmol), α-Chlorophenylacetyl chloride (60 µL, 0.74 mmol) and K_2CO_3 (855 mg, 6.2 mmol). Yield 74 %; TLC R_f = 0.40 (EtOAc/*n*-hexane = 1:4); IR (Neat, ν cm^{-1}): 1738.51, 1682.59, 1638.23, 1409.71; ^{1}H NMR (300 MHz, CD_3OD); δ 7.90-7.85 (m, 2H), 7.44-7.38 (m, 5H), 7.18-7.12 (m, 2H), 5.91 (s, 1H), 4.23-4.03 (m, 2H), 3.63 (s, 3H), 2.82 - 2.77 (m, 2H).; ^{13}C NMR (300 MHz, DMSO-d_6) δ 171.3, 165.3, 162.0, 159.2, 146.7, 135.1, 129.3, 128.8, 128.7, 128.6, 127.1, 125.8, 125.7, 115.9, 115.6, 76.0, 51.4, 43.0, 31.9.; HR-MS (FAB) *m/z* 357.1248 (calcd, for $C_{19}H_{18}FN_2O_4$ [M+H]$^+$, 357.1251); HPLC flow rate: 1 mL/min, relative purity 100%, (t = 13.4 min).

Methyl 3-(2-(4-chlorophenyl)-5-oxo-6-phenyl-5, 6-dihydro-4*H*-1,3,4-oxadiazin-4-yl) propanoate (4c)

The compound **4c** (102 mg) was obtained according to the general procedure from compound **2g** (130 mg, 0.50 mmol), α-Chlorophenylacetyl chloride (103 µL, 0.60 mmol) and K_2CO_3 (690 mg, 5.0 mmol). Yield 54%; TLC R_f = 0.40 (EtOAc/*n*-hexane = 1:4); IR (Neat, ν cm^{-1}): 1738.51, 1682.59, 1635.34, 752.102; ^{1}H NMR (300 MHz, CD_3OD); δ 7.82-7.77 (m, 2H), 7.42-7.36 (m, 7H), 5.89 (s, 1H), 4.24-4.00 (m, 2H), 3.62 (s, 3H), 2.86-2.72 (m, 2H).; ^{13}C NMR (300 MHz, CD_3OD) δ 173.9, 162.0, 149.6, 138.9, 137.0, 131.3, 130.7 (2C), 130.5 (2C), 130.2, 129.8 (2C), 128.8(2C), 78.9, 53.1, 45.5, 33.8.; ESI-MS *m/z*

373 $(M + H)^+$; HR-MS (FAB) *m/z* 373.0959 (calcd, for $C_{19}H_{18}ClN_2O_4$ $[M+H]^+$, 373.0955).

Methyl 3-(6-methyl-2-(4-nitrophenyl)-5-oxo-5, 6-dihydro-4*H*-1,3,4-oxadiazin-4-yl) propanoate (4d)

The compound **4d** (74 mg) was obtained according to the general procedure from compound **2e** (90 mg, 0.33 mmol), 2-chloropropanoyl chloride (50 µL, 0.39 mmol) and K_2CO_3 (455 mg, 3.3 mmol). Yield 69%; TLC R_f = 0.40 (EtOAc/*n*-hexane = 1:4); IR (Neat, ν cm^{-1}): 1737.55, 1686.44, 1635.34, 1523.49; ^{1}H NMR (300 MHz, CD_3OD) δ 8.31 (d, *J* = 9.0 Hz, 2H), 8.09 (d, *J* = 8.7 Hz, 2H), 4.97 (q, *J* = 13.8, 6.9 Hz, 1H), 4.17-4.00 (m, 2H), 3.66 (s, 3H), 2.79 - 2.74 (m, 2H), 1.58 (d, *J* = 6.6 Hz, 3H).; ^{13}C NMR (300 MHz, DMSO-d_6) δ 171.2, 161.6, 148.5, 146.0, 135.3, 127.2 (2C), 123.8 (2C), 71.7, 51.5, 42.9, 31.8, 16.4. ESI-MS *m/z,* 322 $(M + H)^+$; HR-MS (FAB) *m/z* 322.1037 (calcd for $C_{14}H_{16}N_3O_6$ $[M+H]^+$, 322.1039); HPLC flow rate: 1mL/min, relative purity 99 %, (t = 13.2 min).

Methyl 3-(2-(4-fluorophenyl)-6-methyl-5-oxo-5, 6-dihydro-*4H*-1,3,4-oxadiazin-4-yl) propanoate (4e)

The compound **4e** (120 mg) was obtained according to the general procedure from compound **2f** (150 mg, 0.62 mmol), 2-chloropropanoyl chloride (78 µL, 0.74 mmol) and K_2CO_3 (855 mg, 6.2 mmol). Yield 66%; TLC R_f = 0.40 (EtOAc/*n*-hexane = 1:4); IR (Neat, ν cm^{-1}): 1739.48, 1682.59, 1635.34, 1411.64; ^{1}H NMR (300 MHz, CD_3OD) δ 7.93-7.88 (m, 2H), 7.20-715 (m, 2H), 4.91-4.86 (m, 1H), 4.11-3.98 (m, 2H), 3.67 (s, 3H), 2.74 (t, *J* = 6.9 Hz, 2H), 1.57 (d, *J* = 6.7 Hz, 3H).; ^{13}C NMR (400 MHz, DMSO-d_6) δ 171.3, 164.9, 161.7, 147.2, 128.7, 128.6, 126.0, 115.8, 115.5, 71.4, 51.4, 42.7, 31.8, 16.2.; ESI-MS *m/z,* 295 $(M + H)^+$; HR-MS (FAB) *m/z* 295.1097 (calcd, for $C_{14}H_{16}FN_2O_4$ $[M+H]^+$, 295.1094); HPLC flow rate: 1 mL/min, relative purity 93.4%, (t = 12.8 min).

Methyl 3-(2-(4-chlorophenyl)-6-methyl-5-oxo-5, 6-dihydro-4*H*-1,3,4-oxadiazin-4-yl) propanoate (4f)

The compound **4f** (79 mg) was obtained according to the general procedure from compound **2g** (100 mg, 0.39 mmol), 2-chloropropanoyl chloride (49 µL, 0.46 mmol) and K_2CO_3 (838 mg, 3.9 mmol). Yield 65%; TLC R_f = 0.40 (EtOAc/*n*-hexane = 1:4); IR (Neat, ν cm^{-1}): 1739.48, 1683.55, 1633.41, 750.17; ^{1}H NMR (300 MHz, CD_3OD) δ 7.83 (d, *J* = 6.6 Hz, 2H), 7.45 (d, *J* = 6.9 Hz, 2H), 4.90-4.87 (m, 1H), 4.08-3.99 (m, 2H), 3.65 (s, 3H), 2.73 (t, *J* = 6.7 Hz, 2H), 1.55 (d, *J* = 6.6 Hz, 3H).; ^{13}C NMR (400 MHz, DMSO-d_6) δ 171.3, 161.7, 147.1, 135.7, 128.7 (2C), 128.3, 127.9 (2C), 71.5, 51.5, 42.8, 31.9, 16.3.; ESI-MS *m/z* 311 $(M+H)^+$; HR-MS (FAB) *m/z* 311.0794 (calcd for $C_{14}H_{16}ClN_2O_4$ $[M+H]^+$, 311.0799); HPLC flow rate: 1 mL/min, relative purity 100 %, (t = 14.0 min).

2-(1-(2-bromo-2-chloroacetyl)-2-(4-nitrobenzoyl) hydrazinyl)acetamide (5)

A solution of 4-nitrobenzohydrazide (1 mmol) and 2-bromoacetamide (0.90 mmol) in DMF was stirred for 6 h at room temp. After consumption of starting compound, DIC (3 mmol), and Chlorobromoacetic acid (1.2 mmol) was added. The reaction mixture was stirred for another 8 h at room temp. The resulting residue was worked-up with ethyl acetate and purified by column chromatography on silica gel 60 (40-60 µm) using EtOAc/*n*-hexane to give compound 5 in 36% yield.; ^{1}H NMR (300 MHz, DMSO-d_6) δ 11.4 (s, 1H), 8.36 (d, *J* = 8.7 Hz, 2H), 8.15 (d, *J* = 9.0 Hz, 2H), 7.55(s, 1H), 7.23(s, 1H), 7.09 (s, 1H), 4.6 (bs, 1H), 3.9 (bs, 1H).; ESI-MS *m/z* 394 $(M+H)^+$

Synthesis of 2-(6-chloro-2-(4-nitrophenyl)-5-oxo-5, 6-dihydro-4*H*-1,3,4-oxadiazin-4-yl)acetamide (OXA40)

DIEA (3 mmol) was added to a solution of 2-(1-(2-bromo-2-chloroacetyl)-2-(4-nitrobenzoyl) hydrazinyl)acetamide **(5)** (1 mmol) in dry DMF (3 mL). The resulting solution was stirred for 1 h at room temp. The reaction mixture was worked-up with ethyl acetate and purified by column chromatography to afford compound **OXA40** in 78% yield.; ^{1}H NMR (300 MHz, DMSO-d_6) δ 8.38-8.33 (m, 2H), 8.06-8.01 (m, 2H), 7.67 (s, 1H), 7.38 (s, 1H), 7.32 (s, 1H), 4.40 (dd, *J* = 60.7, *J* = 16.2 Hz, 2H); ^{13}C NMR (300 MHz, CD_3OD) δ 171.9, 157.8, 151.8, 145.8, 136.4, 129.6, 129.5, 125.6 (2C), 83.4, 52.5.; ESI-MS *m/z* 313 $(M+H)^+$; HPLC flow rate: 1 mL/min, relative purity 100%, (t = 10.2 min).

SPETRAL DATA OF NEW COMPOUNDS

3a–^{1}H NMR

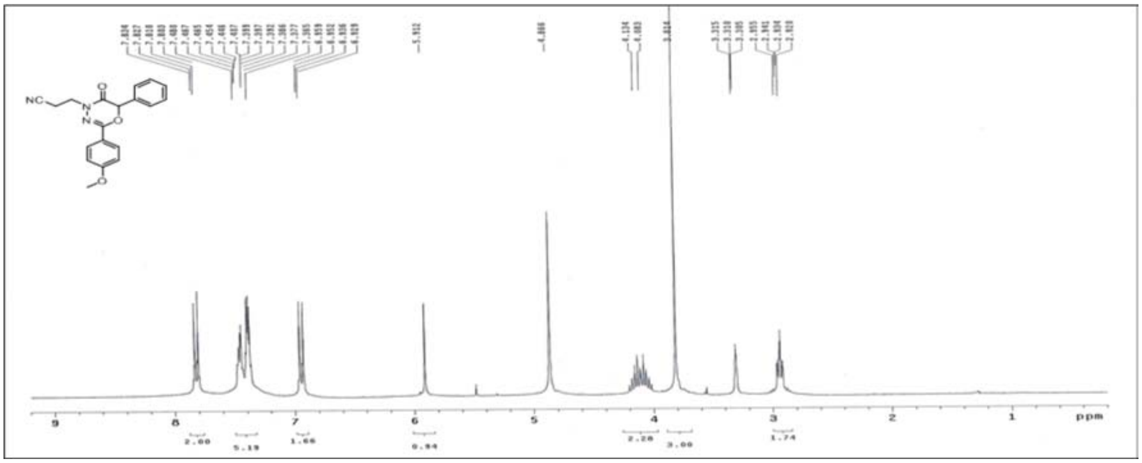

3a–^{13}C NMR

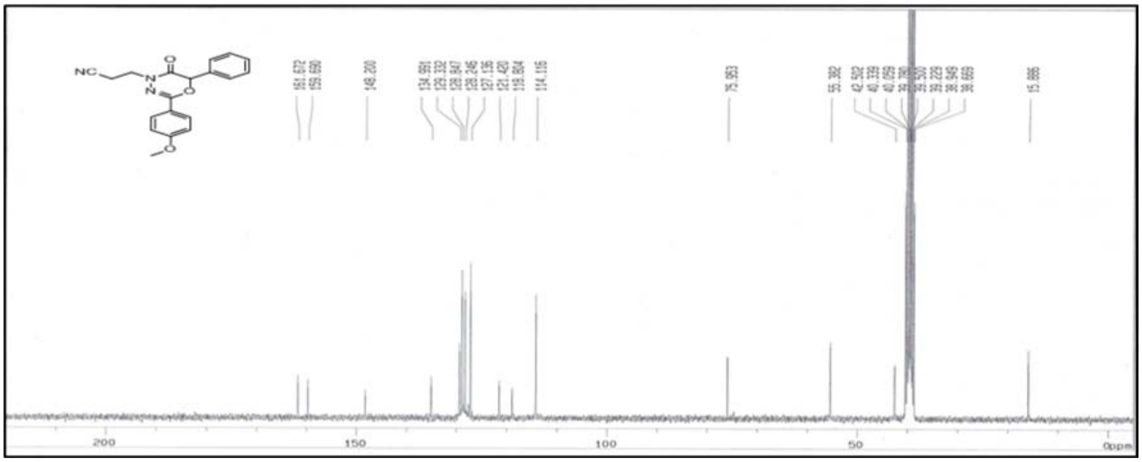

3b–^{1}H NMR

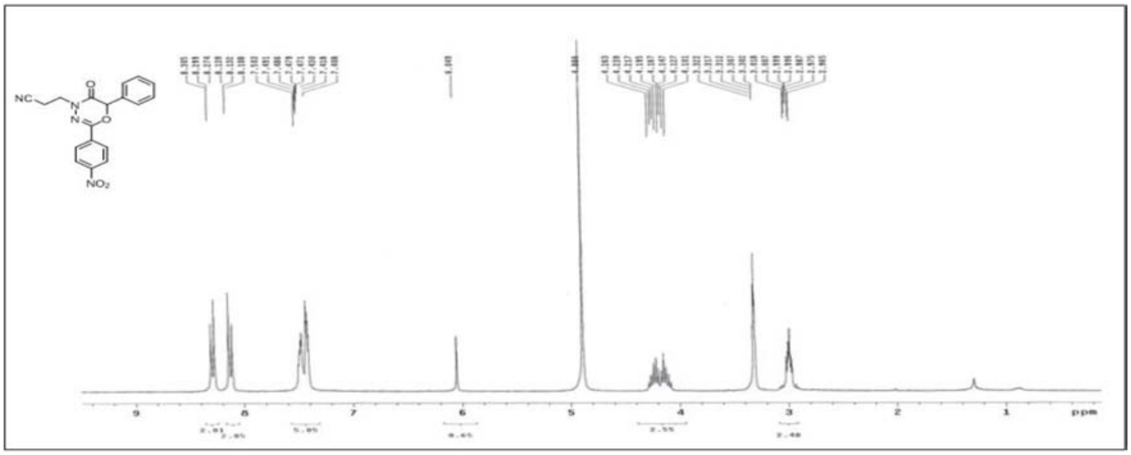

3b–^{13}C NMR

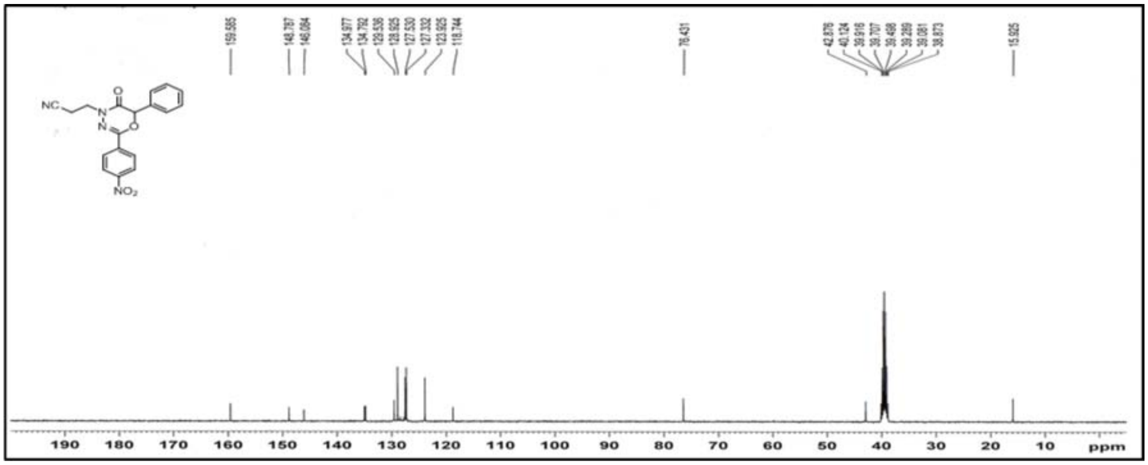

3c–^{1}H NMR

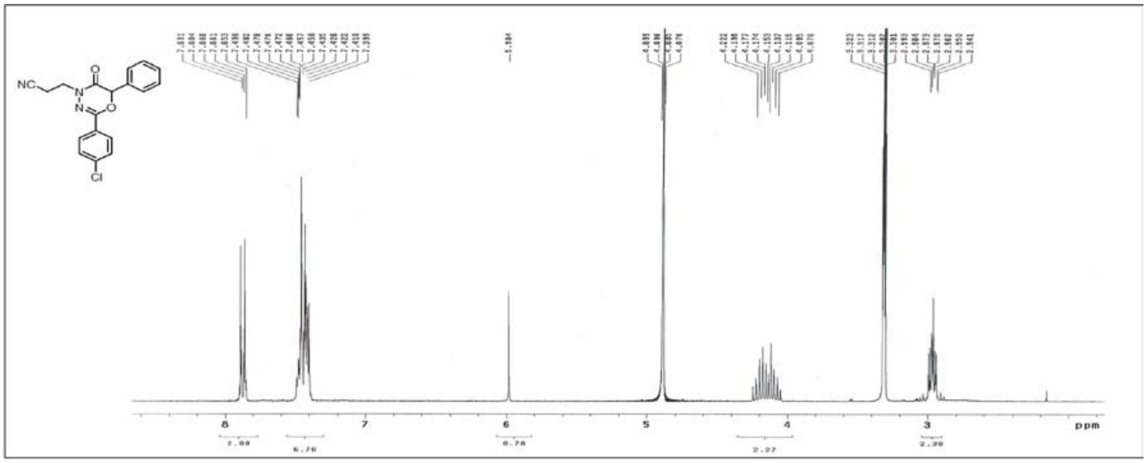

3c–^{13}C NMR

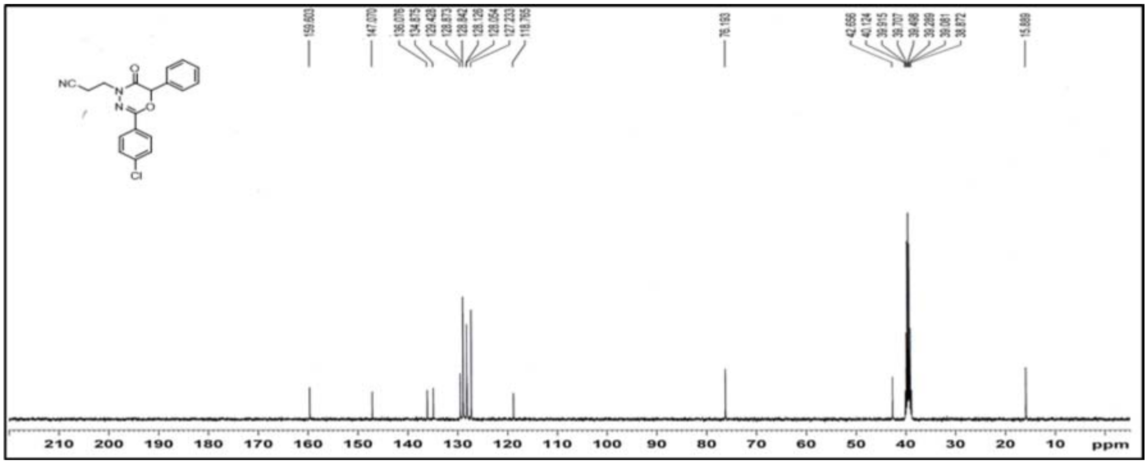

3d–^{1}H NMR

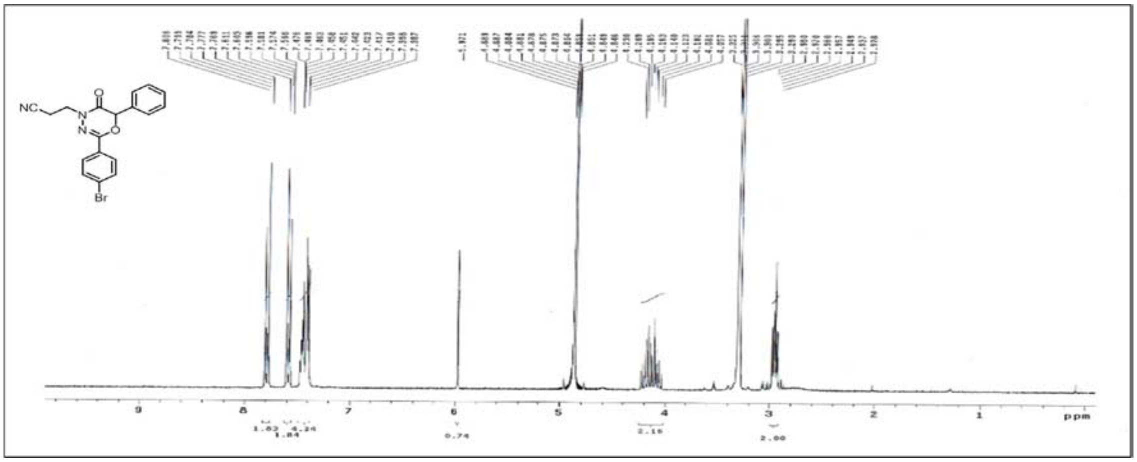

3d–^{13}C NMR

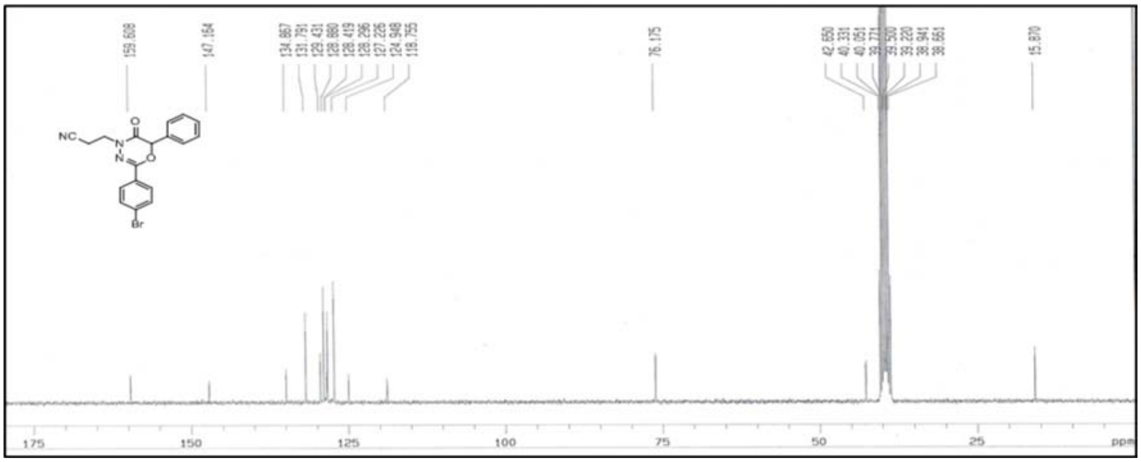

3e–^{1}H NMR

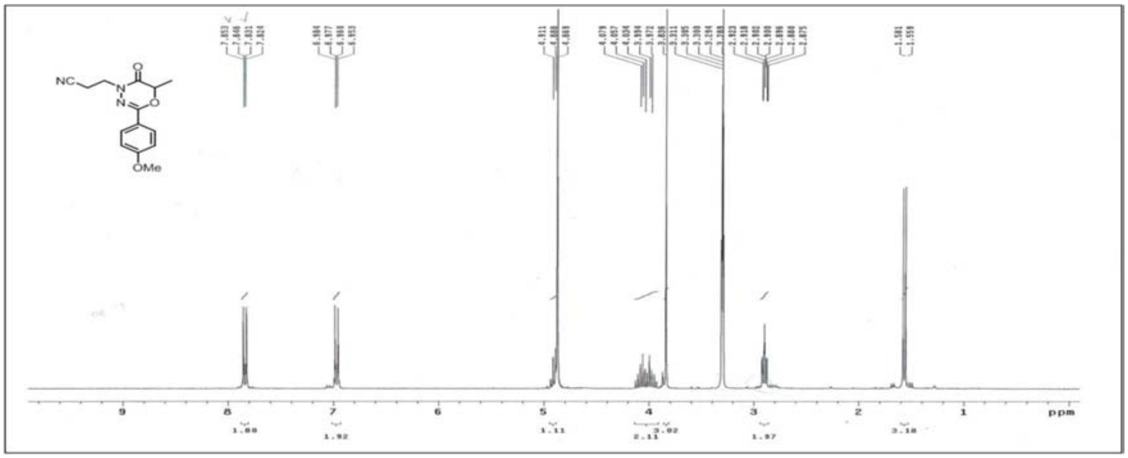

3e–^{13}C NMR

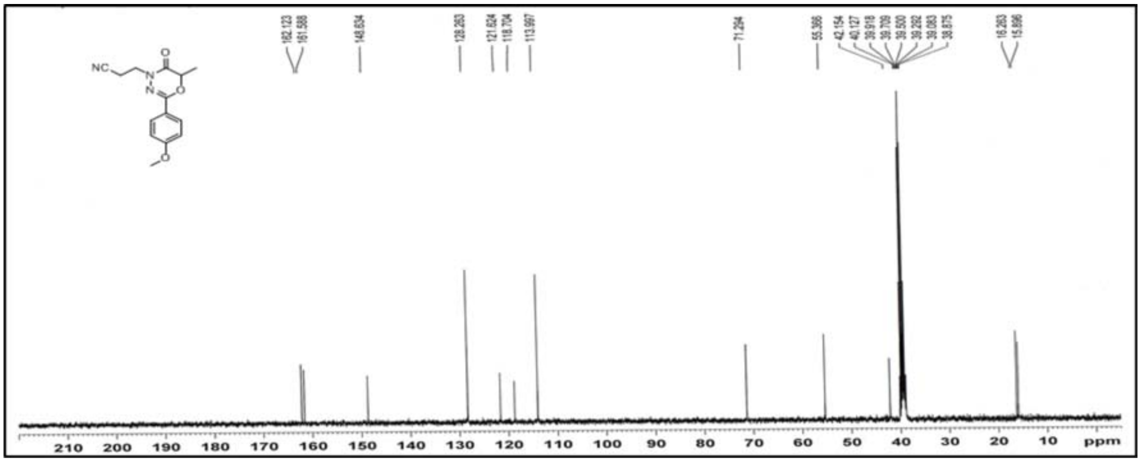

3f–^{1}H NMR

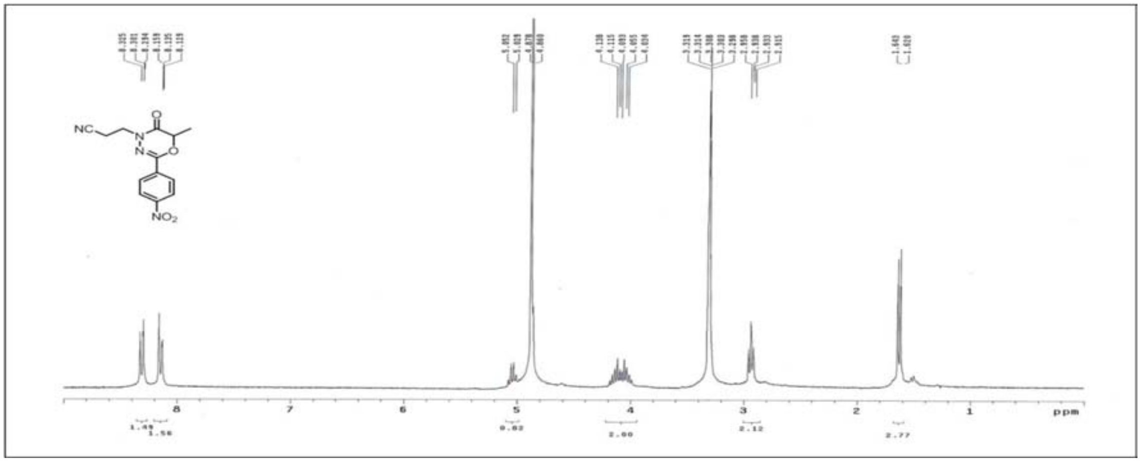

3f–^{13}C NMR

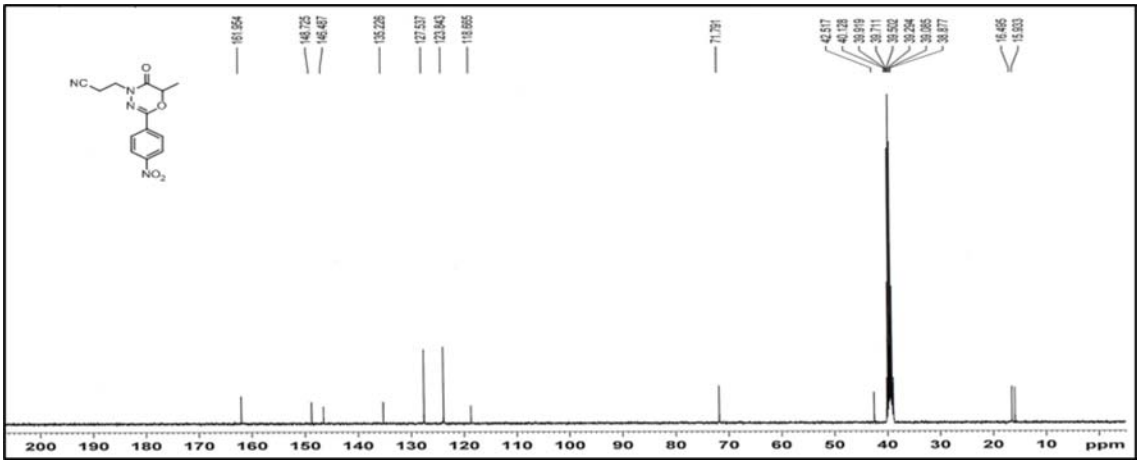

3g–^{1}H NMR

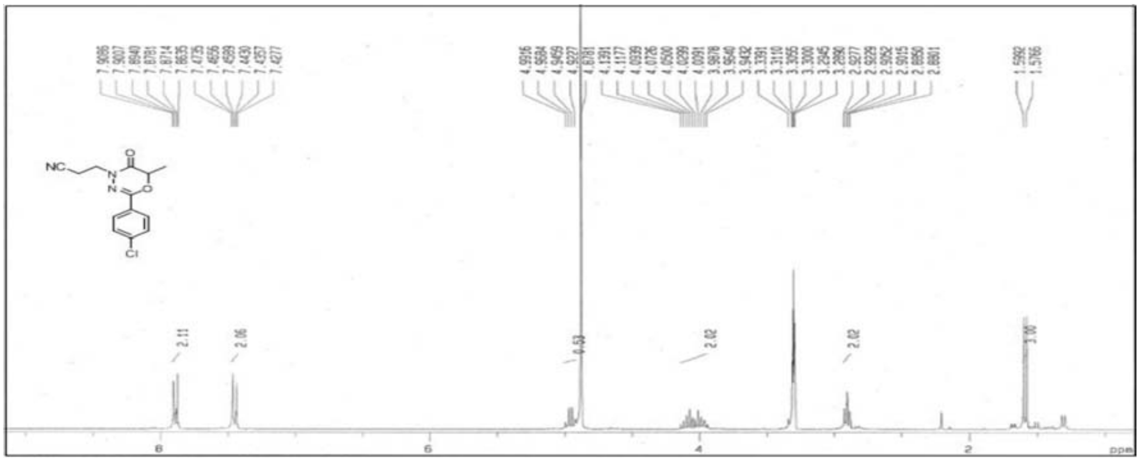

3g–^{13}C NMR

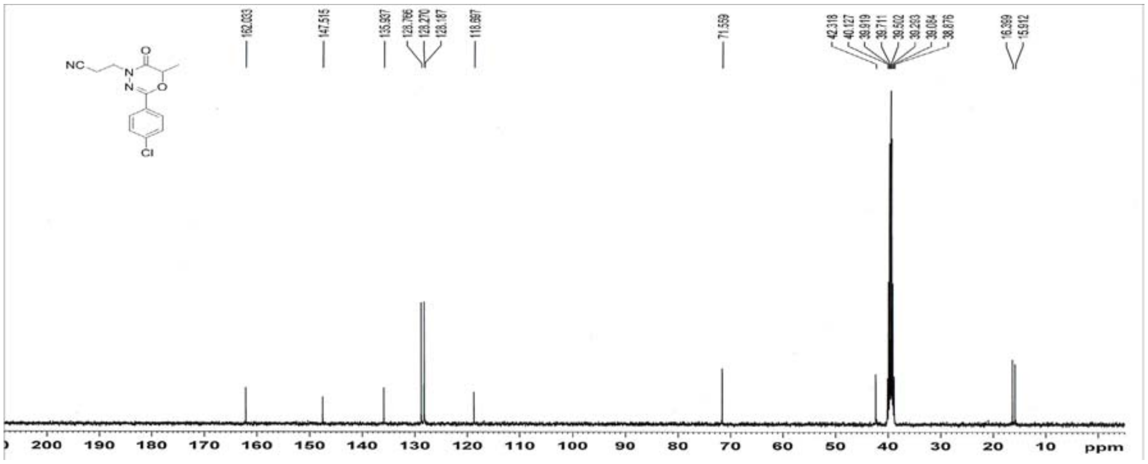

3h–^{1}H NMR

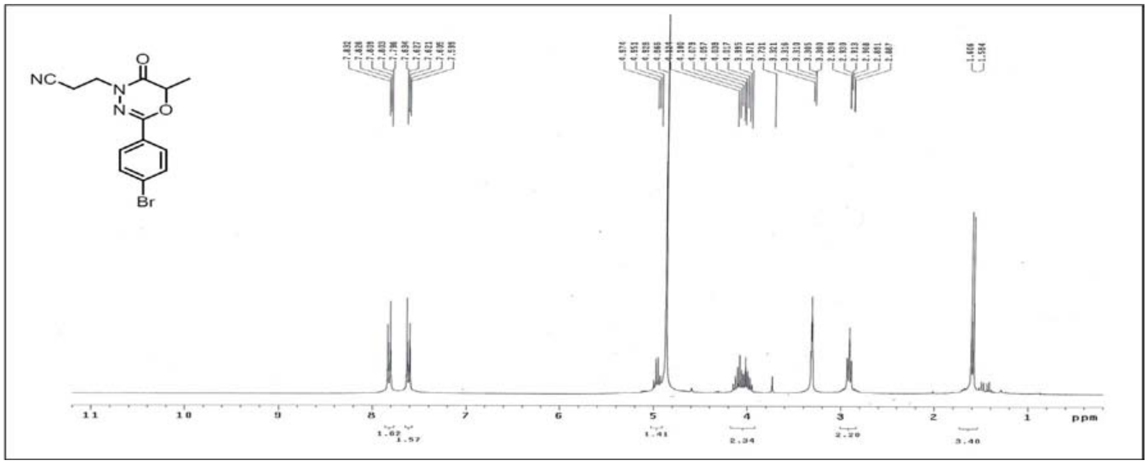

3h–^{13}C NMR

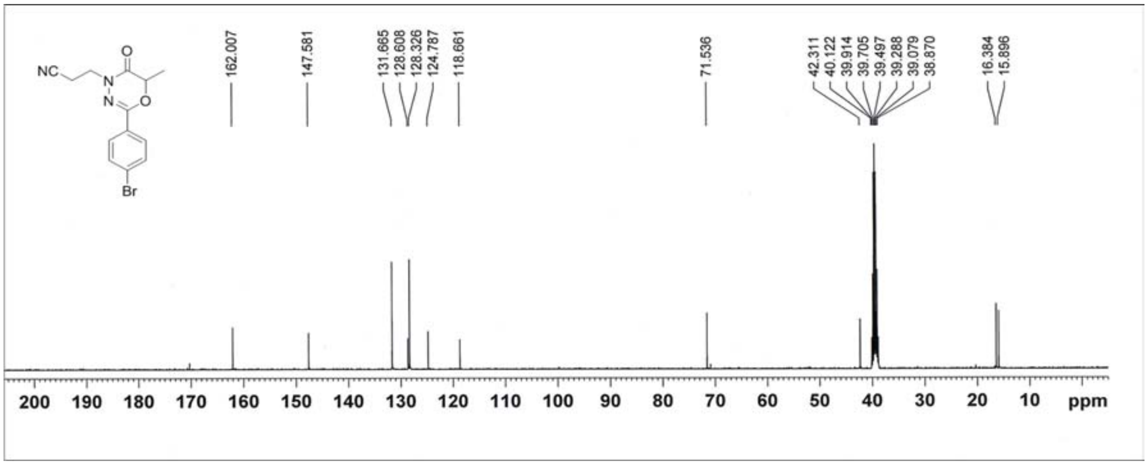

4a–^{1}H NMR

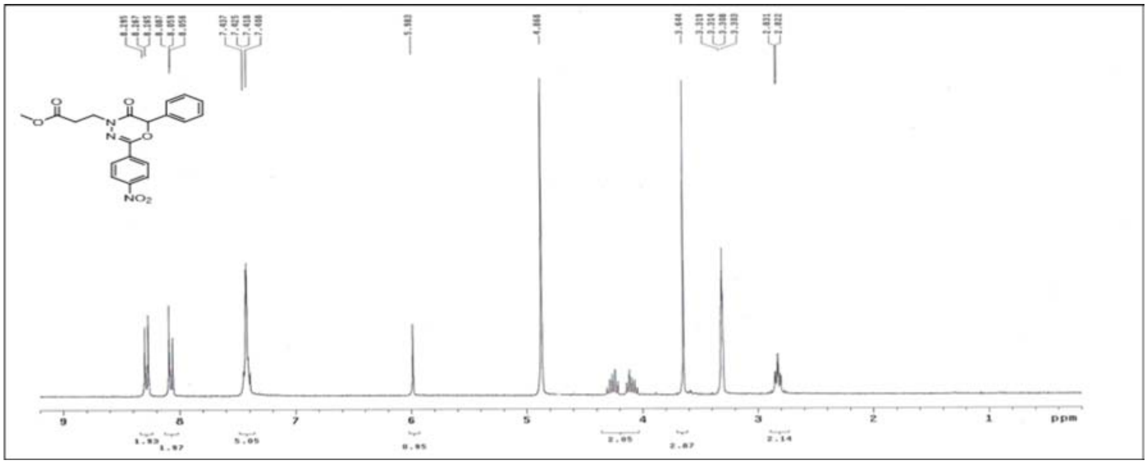

4a–^{13}C NMR

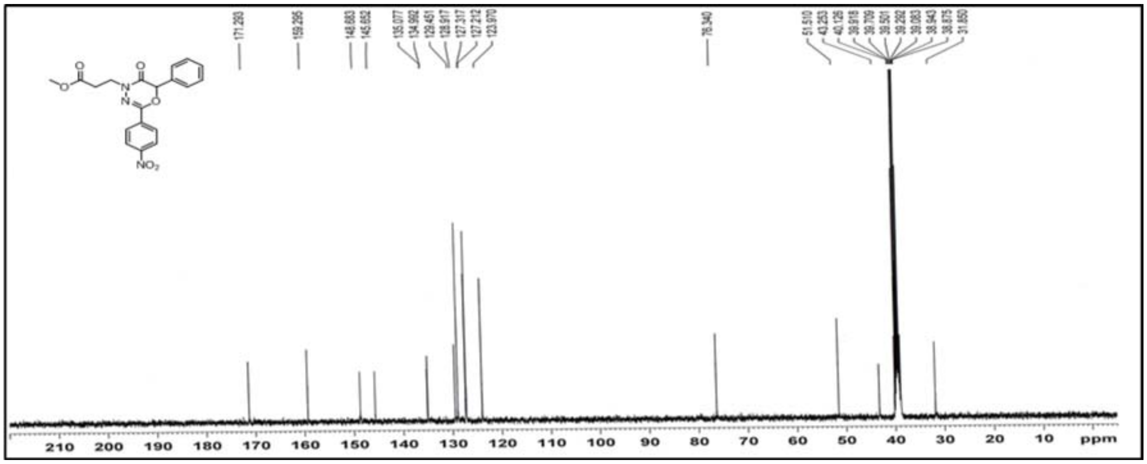

4b–^{1}H NMR

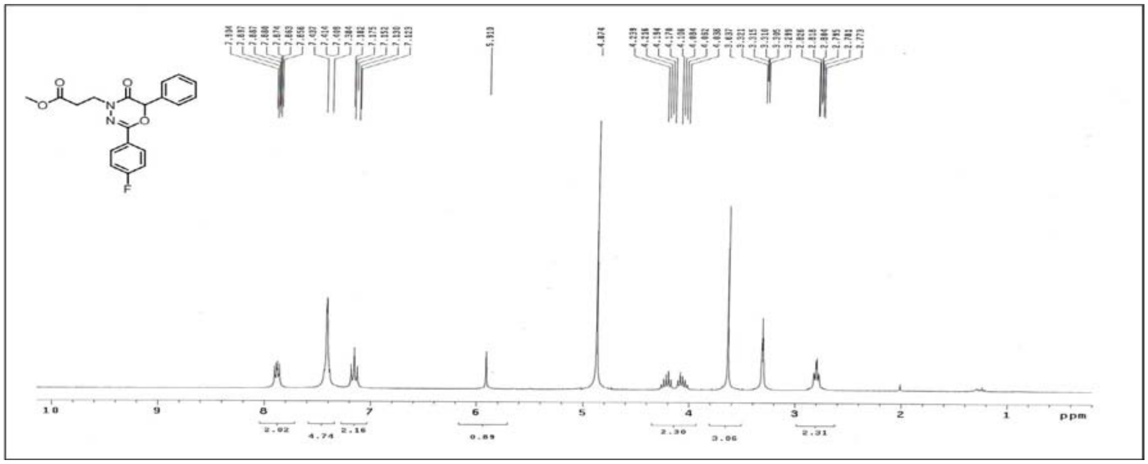

4b–^{13}C NMR

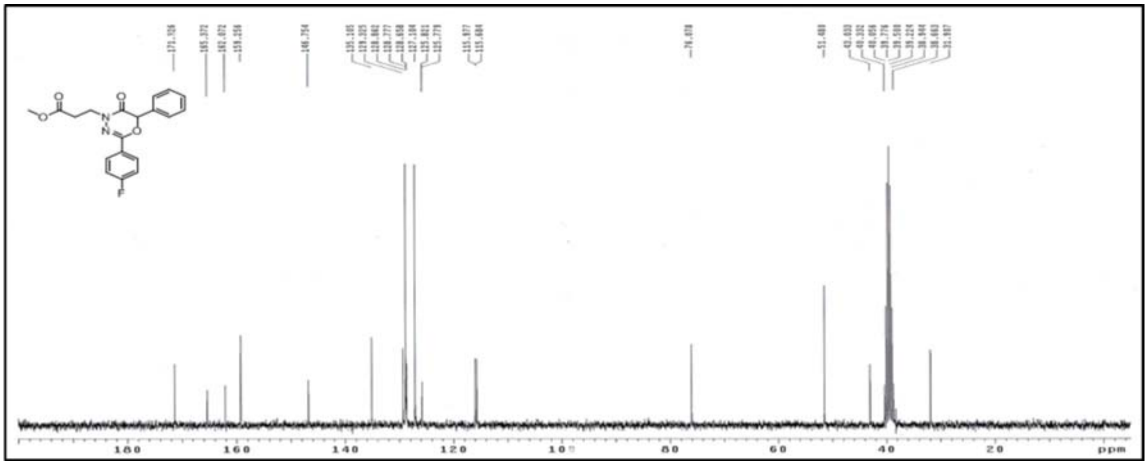

4c–^{1}H NMR

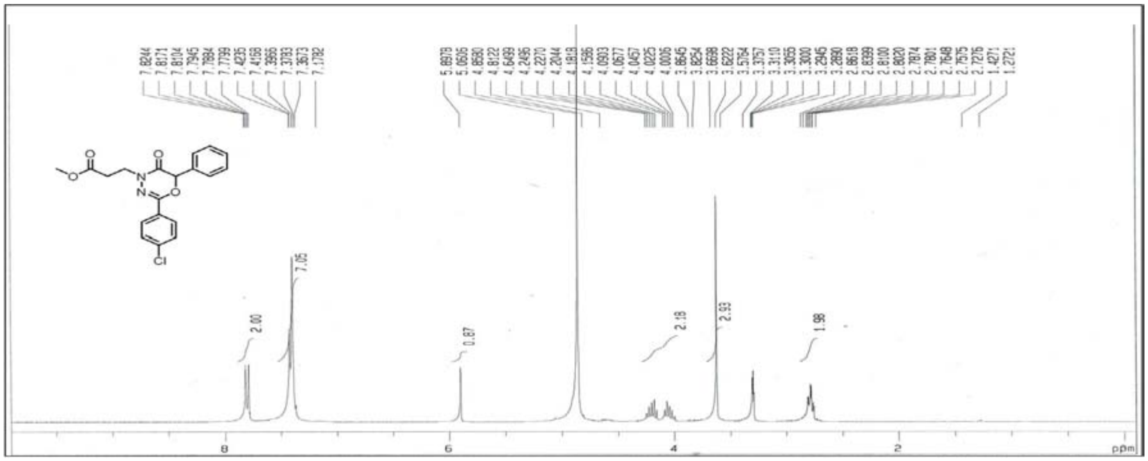

4c–^{13}C NMR

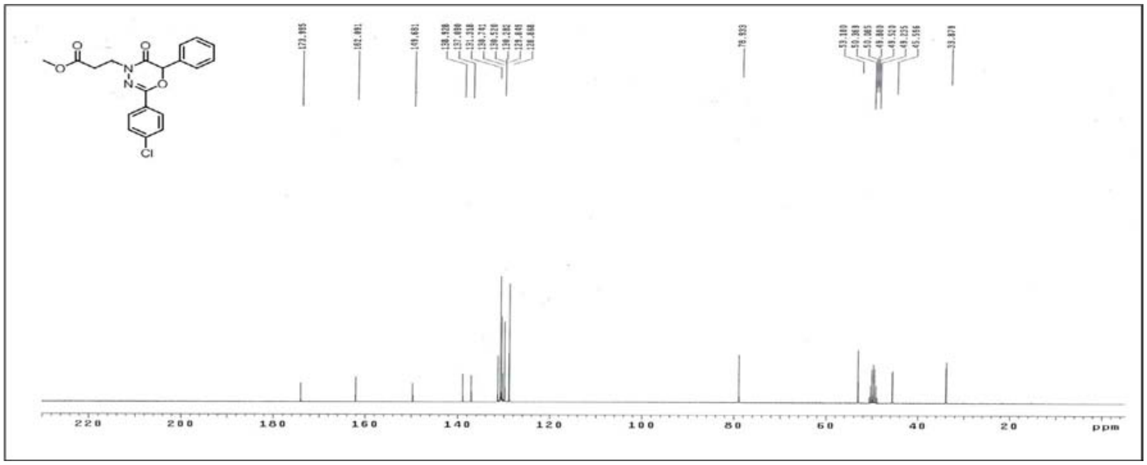

4d–^{1}H NMR

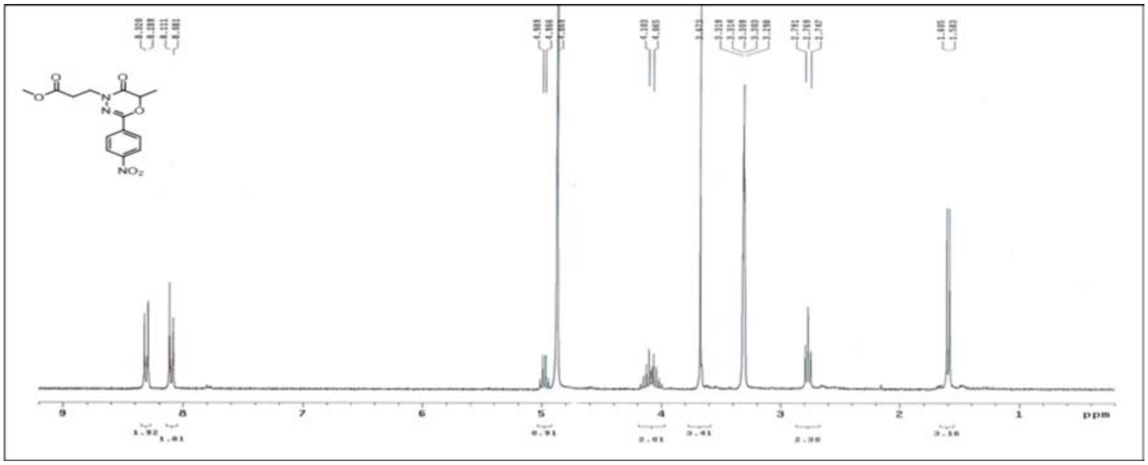

4d–^{13}C NMR

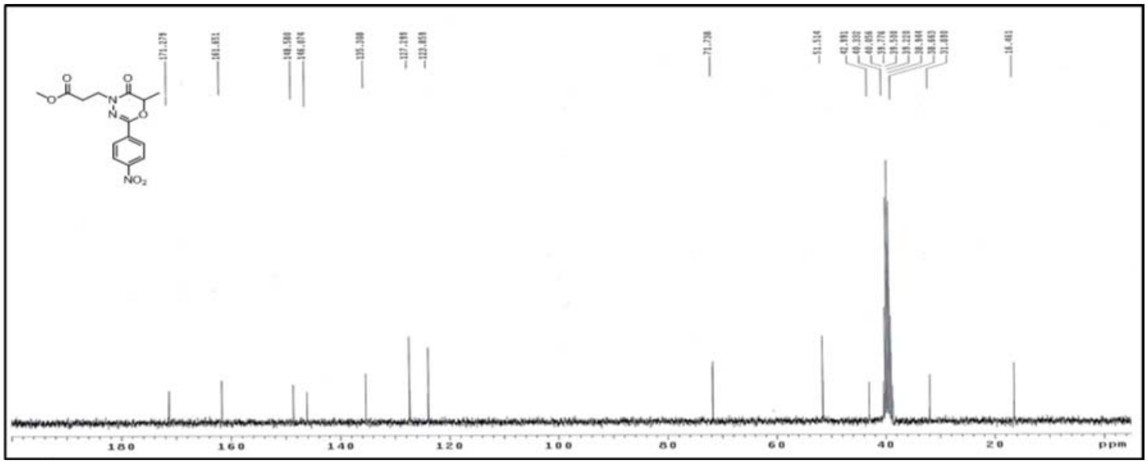

4e–^{1}H NMR

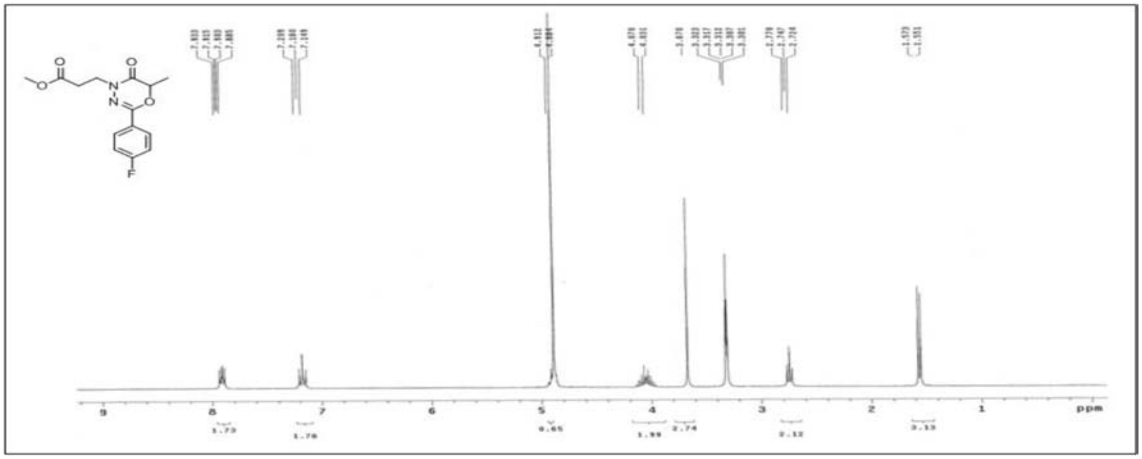

4e–^{13}C NMR

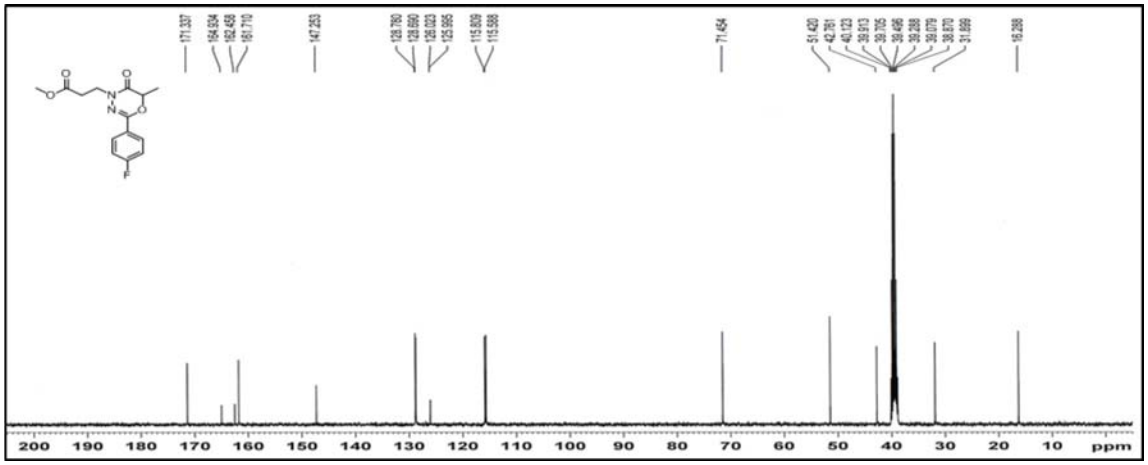

4f–^{1}H NMR

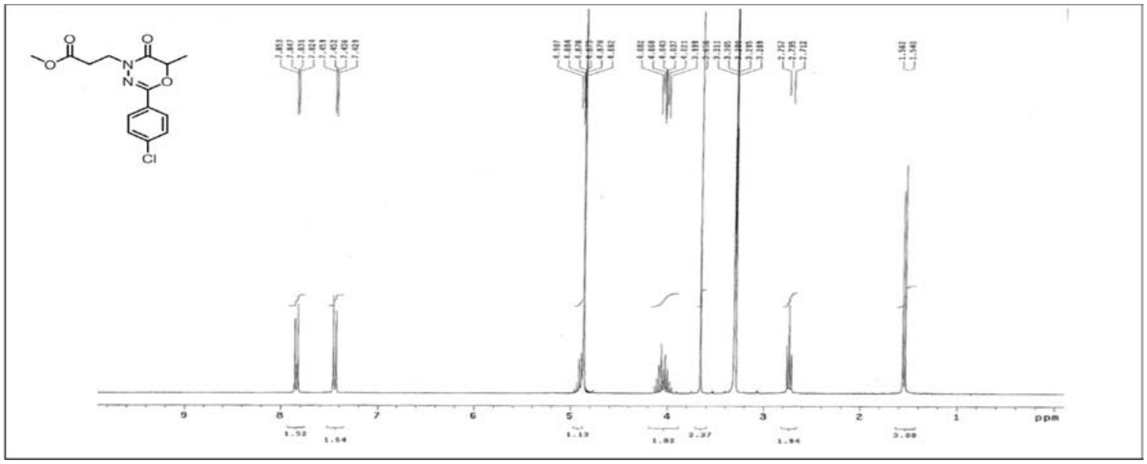

4f–^{13}C NMR

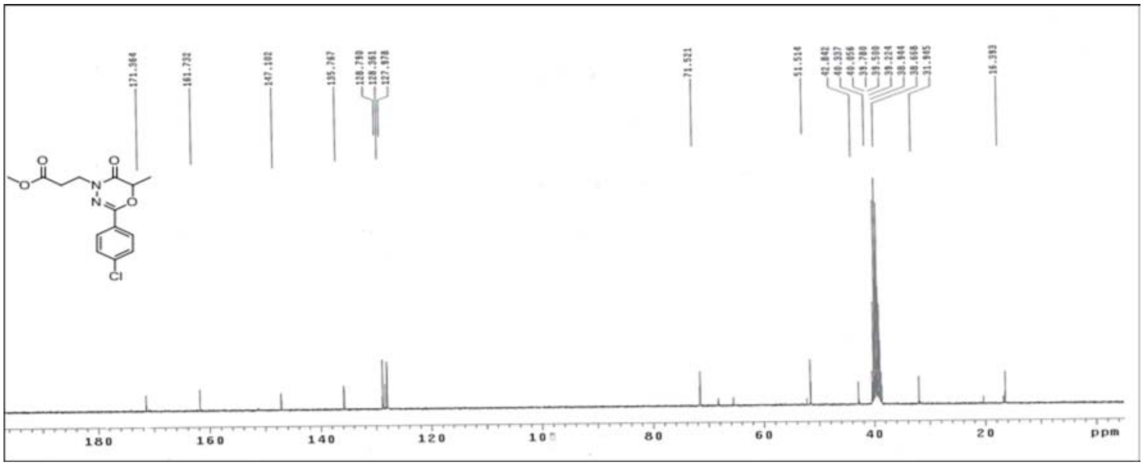

OXA40–^{1}H NMR

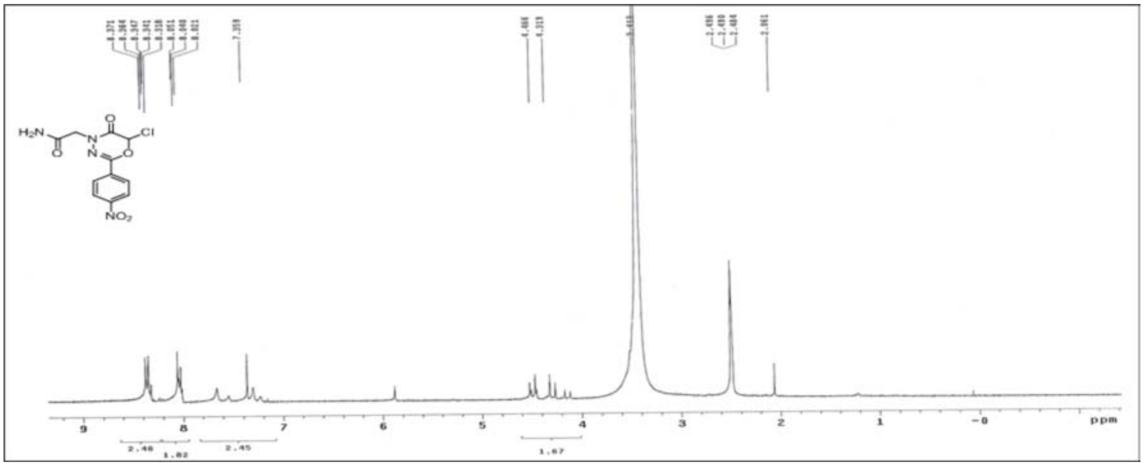

OXA40–^{13}C NMR

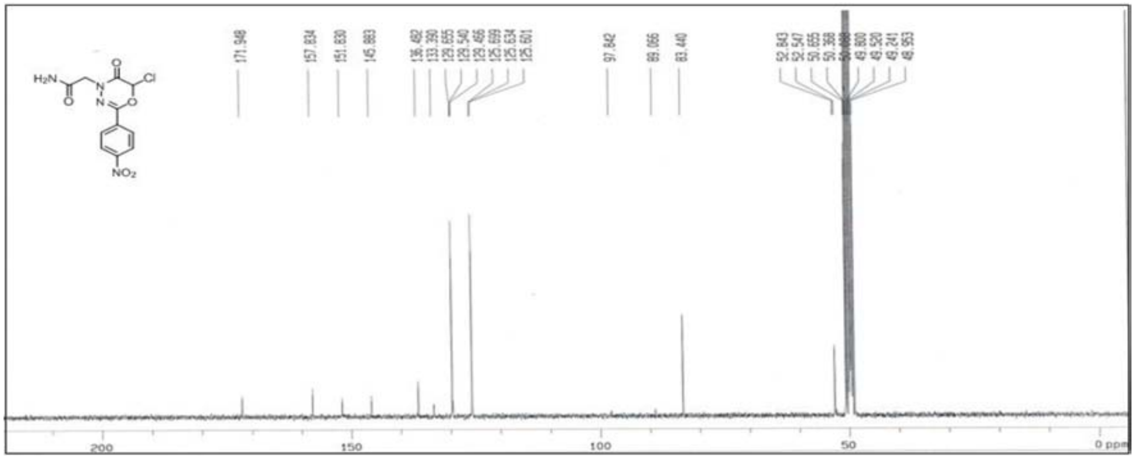

A B

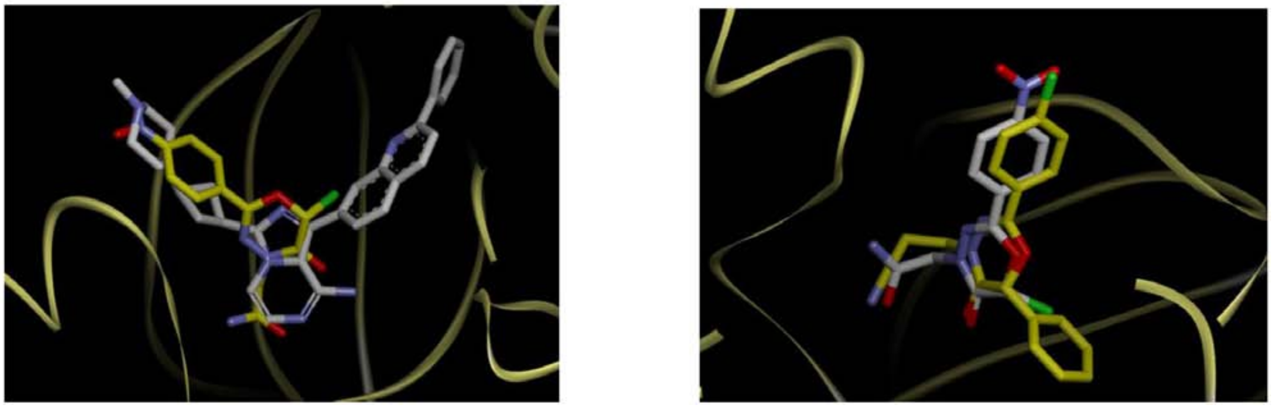

Supplementary Figure S1: Overlaid poses of ligands docked to IGF-1R. A. White: original ligand (PQIP), yellow: **oxa40. B.** The docked structures of **oxa40** (white) and 3c (yellow). The backbones of the compounds are well aligned; the phenyl group fills the hydrophobic pocket.

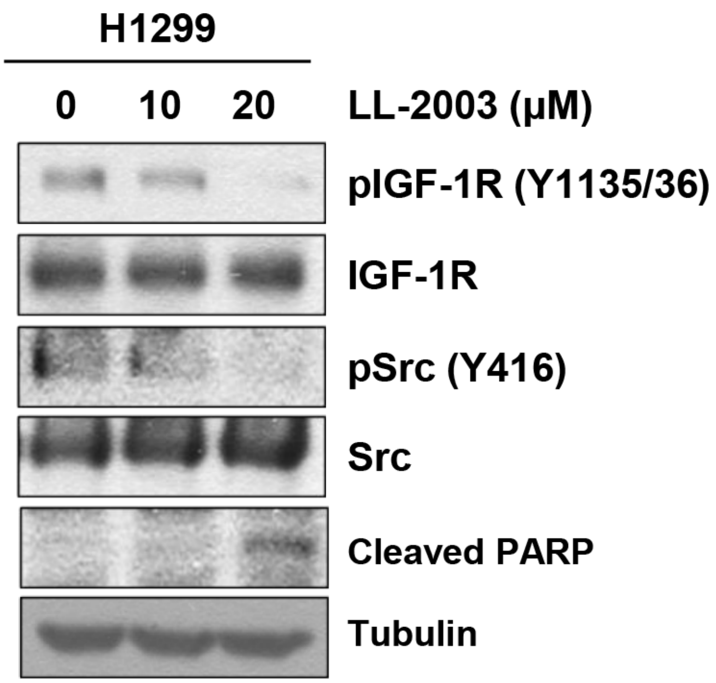

Supplementary Figure S2: Inhibitory effect of LL-2003 on the phosphorylation of IGF-1R and Src. Western blot analysis evaluating the effects of LL-2003 on the indicated protein expression in H1299 cells.

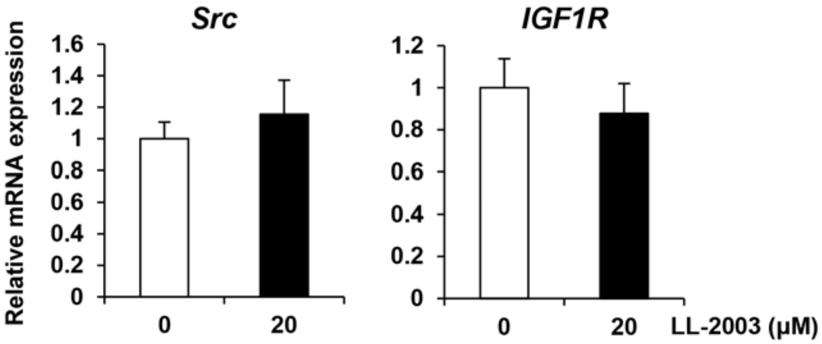

Supplementary Figure S3: Minimal effects of LL-2003 on the mRNA expression of *Src* and *IGF1R*. H1299 cells were treated with vehicle or LL-2003. The relative *Src* and *IGF-1R* mRNA expression was determined by real-time PCR analysis.

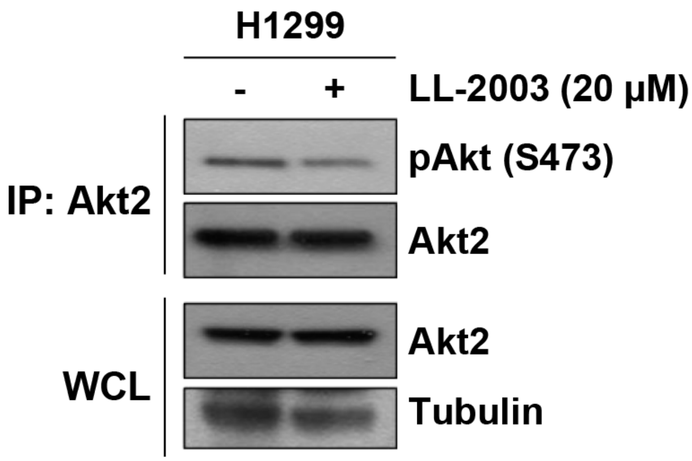

Supplementary Figure S4: Suppression of Akt2 phosphorylation by treatment with LL-2003. Whole cell lysates obtained from H1299 cells treated with vehicle or LL-2003 (20 μM) were immunoprecipitated with anti-Akt2 antibodies, and Western blot analysis using an anti-pAkt (S473) antibody was followed.

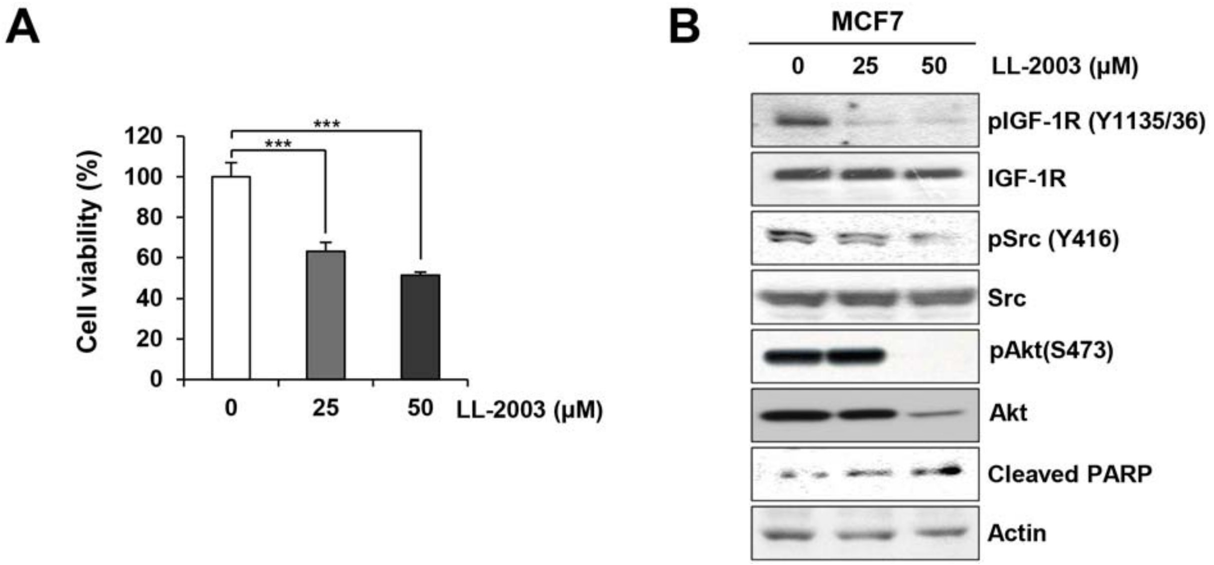

Supplementary Figure S5: Effects of LL-2003 on cell viability and the phosphorylation of IGF-1R and Src in MCF7 human breast cancer cells. A. The MTT assay for evaluating the effect of LL-2003 on MCF7 cell viability. **B.** Western blot analysis evaluating the effects of LL-2003 on the indicated protein expression in MCF7 cells.

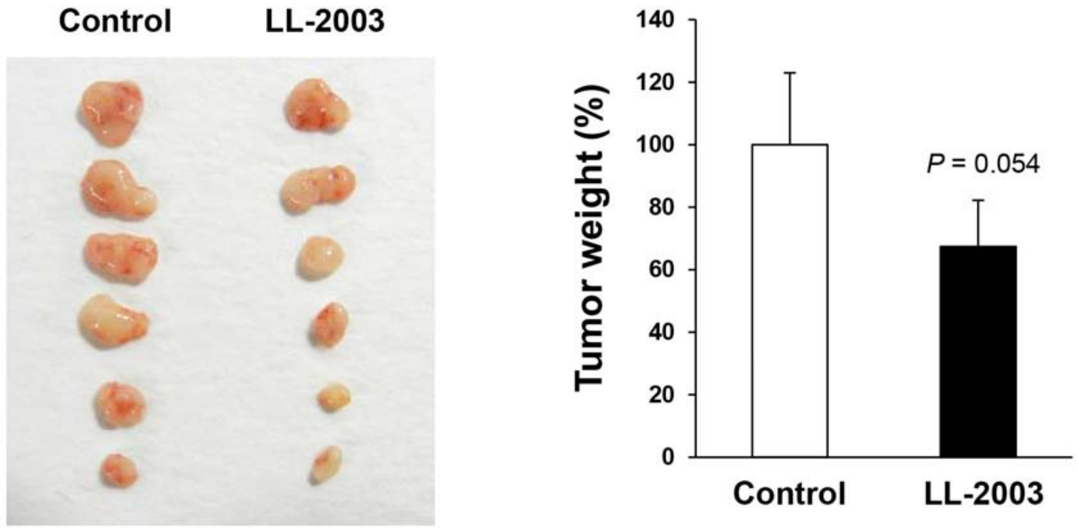

Supplementary Figure S6: Antitumor effect of LL-2003 in a tumor xenograft model. *Left*. Representative images of tumors of each group. *Right*. The effects of LL-2003 on xenograft tumor weight.

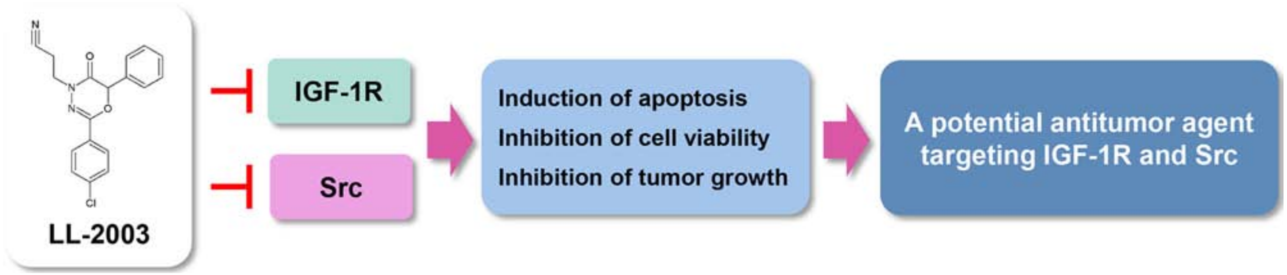

Supplementary Figure S7: Schematic model for the main findings of the present study.

Supplementary Table S1: Docking scores of selected ligands in IGF-1R and Src binding sites

Compound #	IGF-1R			Src		
	Surflex-Dock total score (-logK_d)	Crash	Polar	Surflex-Dock total score (-logK_d)	Crash	Polar
**OXA40**	5.5535	−0.6493	3.1372	5.1727	−0.6179	3.6130
**3b**	6.7235	−1.1729	0.7479	7.5363	−0.7665	1.9466
**3c (LL-2003)**	7.5946	−1.3665	2.3860	6.4196	−1.2135	0.7870
**3d**	6.8658	−0.5649	0.4341	6.3901	−0.9074	0.6846
**4c**	7.7141	−0.5602	1.7115	7.0490	−1.6231	0.5933
